# In‐depth multiomic characterization of the effects of obesity in high‐fat diet‐fed mice

**DOI:** 10.1002/2211-5463.13788

**Published:** 2024-03-13

**Authors:** Boping Li, Juanjuan Chen, Xiaobin Ou, Xiuli Liu, Zaoxu Xu, Xuesong Xiang, Yan Yang, Qi Wang

**Affiliations:** ^1^ Gansu Key Laboratory of Protection and Utilization for Biological Resources and Ecological Restoration in Longdong Longdong University Qingyang China; ^2^ College of Medicine Longdong University Qingyang China; ^3^ Cuiying Biomedical Research Center Lanzhou University Second Hospital Lanzhou China; ^4^ College of Life Sciences and Technology Longdong University Qingyang China; ^5^ Element Nutrition of National Health Commission, National Institute of Nutrition and Health China CDC Beijing China; ^6^ Department of Endocrinology and Metabolism Lanzhou University Second Hospital Lanzhou China

**Keywords:** colonic gene expression, gut microbiome, high‐fat diet‐fed obesity, liver‐specific gene expression, metabolomics, multiomics

## Abstract

High‐fat diet (HFD)‐fed mice have been widely used in the clinical investigation of obesity. However, the long‐term effect of HFD on gut microbiota and metabolites, plasma and liver metabolomics, colonic and liver transcriptomics remain largely unknown. In this study, 6‐week‐old C57BL/6J male mice fed with HFD for 14 weeks showed increased obesity‐related indexes including alanine aminotransferase, aspartate aminotransferase, total cholesterol, total triglyceride, free fatty acids, lipopolysaccharides, IL‐6, and TNFα. Furthermore, microbial diversity and richness were also significantly decreased. In the colon, genes involved in tryptophan metabolism, PPAR signaling pathway, cholesterol metabolism, and lipid localization and transport, were upregulated. While in the liver, MAPK signaling and unsaturated fatty acid biosynthesis were upregulated. Metabolomic analyses revealed decreased levels of glycerophospholipids and fatty acyl, but increased amino acids, coenzymes and vitamins, and organic acids in the colon, suggesting high absorption of oxidized lipids, while acyl‐carnitine, lysophosphatidylcholine, lysophosphatidylethanolamine, and oxidized lipids were reduced in the liver, suggesting a more active lipid metabolism. Finally, correlation analyses revealed a positive correlation between gut microbiota and metabolites and the expression of genes associated with lipid localization, absorption, and transport in the colon, and nutrients and energy metabolism in the liver. Taken together, our results provide a comprehensive characterization of long‐term HFD‐induced obesity in mice.

## Graphical abstract

Using high‐fat diet‐fed mice, we carried out an in‐depth multiomic characterization of the effects of obesity and showed changes and associations among gut microbiota, colonic, plasma, and liver metabolites, and colonic and liver gene expression. Significantly altered gut microbiota was correlated with lipid transport genes in the colon and lipid metabolism genes in the liver.

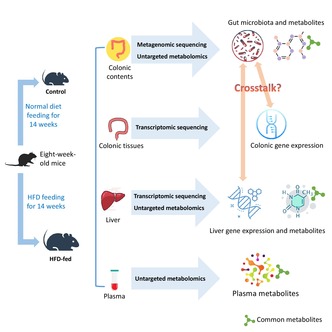

**Abbreviations**
ABC: ATP‐binding cassette
ALT: alanine aminotransferase
AMPK: AMP‐activated protein kinase
AST: aspartate aminotransferase
BP: biological processes
BW: body weight
CC: cellular components
DEGs: differentially expressed genes
DHA: docosahexaenoic acid
DNA: deoxyribonucleic acid
ELISA: enzyme‐linked immunosorbent assay
EPA: eicosapentaenoic acid
FFA: free fatty acids
FPKM: fragments per kilobase million
GO: Gene Ontology
H&E: hematoxylin and eosin
HFD: high‐fat diet
HUMAnN: HMP Unified Metabolic Analysis Network
IL‐6: interleukin 6
IPA: indolepropionic acid
KEGG: Kyoto Encyclopedia of Genes and Genomes
LPA: lysophosphatidic acid
LPC: lysophosphatidylcholine
LPS: lipopolysaccharides
MAPK: mitogen‐activated protein kinase
Metaphlan: metagenomic phylogenetic analysis
MF: molecular functions
NAFLD: nonalcoholic fatty liver disease
PAS: periodic acid‐Schiff
PCoA: principal coordinate analysis
PERMANOVA: permutational multivariate analysis of variance
PPAR: peroxisome proliferator‐activated receptor
RNA: ribonucleic acid
SCFAs: short‐chain fatty acids
SM: sphingomyelin
SPF: specified pathogen free
T2D: type 2 diabetes mellitus
TC: total cholesterol
TG: total triglyceride
TMAO: trimethylamine *N*‐oxide
TNFα: tumor necrosis factor α
UA: uric acid
UPLC‐MS/MS: ultra‐performance liquid chromatography–tandem mass spectrometry

Obesity is globally pandemic and its prevalence is increasing worldwide mostly in Western countries due to a sedentary lifestyle and consumption of high‐fat/high‐sugar diets [1], which represents a major health challenge because it substantially increases the risk of diseases such as T2D, fatty liver disease, hypertension, myocardial infarction, stroke, dementia, osteoarthritis, obstructive sleep apnoea and several cancers [2]. However, obesity prevention and treatment strategies have not been successful in the long term so far, indicating the need for more studies to better understand these diseases and related complications. Diet‐induced obesity (DIO) animal models are widely used in clinical investigation because it can reproduce human obesity [3]. High‐fat diet (HFD), which consisting of at least 35% of total calories is consumed from fats, is often used to induce rodent obesity in animal research because of the direct relationship between the amount of dietary fat and the degree of obesity [4]. Rodent diet with 60 kcal% fat (D12492) has become a standard food for research on obesity, diabetes, and metabolic syndrome. Thus, in this study, we used D12492 as high‐fat diet to induce mouse obesity for further study.

Gut microbiota is involved in the maintenance of energy homeostasis and metabolism. Multiomics studies such as shotgun metagenomic sequencing, transcriptomics, and metabolomics are promising approaches in accurately characterizing metabolic diseases such as obesity. Accumulating evidence has demonstrated changes in the gut microbiome and its metabolites in obesity [5]. Wang *et al*. [6] showed that a refined HFD increased gut microbial diversity and short‐chain fatty acids (SCFAs), which increased the abundance of Desulfovibrionaceae and *Mucispirillum* but reduced that of *Lactobacillus*, *Bifidobacterium*, *Akkermansia*, *Faecalibaculum*, and *Blautia*. Jo *et al*. [7] identified changes in gut microbiota‐metabolomic signatures, wherein a 60% fat diet for 8 weeks significantly increased the relative abundance of Firmicutes, including the genera *Lactococcus*, *Blautia*, *Lachnoclostridium*, *Oscillibacter*, *Ruminiclostridium*, *Harryflintia*, *Lactobacillus*, *Oscillospira*, and *Erysipelatoclostridium*, whereas significantly decreased that of Bacteroidetes. Chen *et al*. [8] investigated key metabolites important in the regulation of metabolic traits, including l‐kynurenine, methyl palmitate, and uric acid, as well as, fatty acid biosynthesis, phenylalanine metabolism, propanoate metabolism, and valine, leucine, and isoleucine degradation in obese individuals. These results separately revealed changes in the gut microbiota and microbial metabolites in HFD‐induced obesity. However, the underlying mechanism of gut microbiome and metabolites on obesity remains unclear. Changes in colonic transcriptome was observed in HFD‐induced obesity [9] and human [10], suggesting that gut microbiota and metabolites may affect colonic gene expression. Qin *et al*. [11] suggested that the gut microbiome stimulates a reprogramming of the enhancer landscape in the colon, with downstream effects on transcription factors under specific dietary exposures. Kim *et al*. [12] have identified 17 leptin‐associated genes including *Peli3*, *Creb1*, and *Enpp2* and 4 insulin‐associated genes including *Centg1* are reported to inversely responded to HFD and obesity. However, the relationship among gut microbiome, microbial metabolites, and colonic gene expression in HFD‐induced obesity mice were not reported.

Except for causing changes in the intestinal microenvironment and colonic gene expression, some gut microbiota‐derived metabolites could go across the gut barrier and enter into the bloodstream to play a role in various organs such as liver [13]. Plasma metabolites have been revealed to be predictive of ectopic fat in the liver [14] and plasma lipid metabolites have been reported as potential biomarkers for identifying individuals at risk of obesity‐induced metabolic complications [15]. Pan *et al*. [16] identified multiple obesity‐related metabolites and metabolomic signatures that were strongly associated with type 2 diabetes in Chinese adults. In addition, the associations between gut microbiota including *Blautia*, *Dorea*, *Ruminococcus*, and *SHA‐98* and BMI‐predictive plasma metabolites, including glutamate and branched‐chain amino acids have been reported [17]. The liver is a key metabolic organ that maintains intrahepatic lipid and glucose metabolism homeostasis [18]. Investigations of the liver transcriptome and metabolism are thought to be of great significance in metabolic diseases and the transcriptional regulatory landscape in the liver induced by HFD was reported [19]. Quintana‐Castro *et al*. [20] reported that obesity is involved in differentiated or tissue‐specific expression of Cd36 mRNA to favor free fatty acids transportation and lipid storage. Ma *et al*. [21] found that the upregulated differentially expressed genes (DEGs) in HFD‐induced nonalcoholic fatty liver disease (NAFLD) were mainly focused on lipid metabolism and synthesis. Wang *et al*. investigated alterations in liver gene expression after chitosan treatment in HFD‐fed mice and indicated that increased *Mups*, *Lcn2*, *Gstm3*, and *CYP2E1* expressions clearly indicated HFD induced lipid metabolism disorder and oxidative damage [22]. Chen *et al*. [23] revealed one lncRNA, which is potentially regulated by PPARα, regulates cellular cholesterol levels in HFD‐fed mice.

In summary, the role of the gut microbiota and microbial metabolism, colonic transcriptome, plasma metabolites, liver transcription and metabolism have been investigated in HFD‐induced obesity and obesity‐related diseases has been studied separately. However, there is a lack of a comprehensive exploration to identify a clear link between these different multiomic aspects in the same HFD‐fed mice. This study was aimed at exploring the characteristics and changes in the gut microbiota and microbial metabolites, colonic and liver gene expression, and liver and plasma metabolites, as well as the complex relationships and connection among these multiomics in long‐term HFD‐induced obesity mouse model.

## Materials and methods

### Ethical standards

The animal experiments were conducted in accordance with the guidelines prescribed by the Chinese Association for Laboratory Animal Sciences and were approved by the Ethical Approval for Research Involving Animals of Lanzhou University Second Hospital (Approval No. D2022‐135) on 23 May 2022.

### Study design

This study was aimed to investigate the multiomic characterization including gut microbiota, colonic and liver gene expression, fecal, liver and plasma metabolome between the HFD‐fed mice and control mice. The experimental unit is group of animals.

### Sample size

Eight mice were included in each group for animal experiment. After the experiment, totally 64 samples including 16 colonic contents, 16 blood samples, 16 liver tissues, and 16 colonic tissues from both HFD‐fed and control mice were collected for multiomic detection and analysis.

### Inclusion and exclusion of criteria

All the eight mice in the HFD‐fed and control group were included for sample collection and the following multiomic test and analysis.

### Experimental animals

Eight‐week‐old male C57BL/6J mice were purchased from Lanzhou Veterinary Research Institute, Chinese Academy of Agricultural Sciences (Lanzhou, China) and were acclimatized in a temperature‐, humidity‐, and light‐controlled ((22 ± 2) °C, (60 ± 5)%, 12‐h light–dark cycle) SPF animal laboratory for 3 days.

### Randomization

The mice were randomly divided into two groups, and each mouse was randomly numbered with 1–8. The HFD‐fed group were fed with HFD (D12492, 60% energy by fat) for 14 weeks, while the control mice were given normal diet (10% fat). Both groups of mice were given free drinking water. BW were recorded every week.

### Blinding

At the terminal of 14 weeks of HFD feeding, the mice were over‐anesthetized to sacrifice to collect biological samples by B. Li *et al*. in Longdong University and no distinction mark were made between two groups. Colonic contents were collected for shotgun metagenomic sequencing and untargeted metabolomic analysis. Colon tissues were collected for transcriptome. Blood samples were collected for untargeted metabolism and biochemical indexes detection. Liver tissues were collected for transcriptome, untargeted metabolome and biochemical indexes examination. The heart, liver, spleen, lungs, kidneys, and stomach tissues were collected for histopathological examination.

The collected samples were quickly stored at −80 °C or liquid nitrogen until all the samples were collected. Samples were then transported on dry ice to laboratory for undifferentiated multiomic and biochemical testing by professional operators without being told the details of sample grouping. The obesity‐related biomedical indexes including aspartate aminotransferase (ATS), alanine transaminase (ALT), total cholesterol (TC), total triglyceride (TG), free fatty acids (FFA) in liver and blood were examined with enzyme‐linked immunosorbent assay (ELISA). Additionally, the obesity‐related cytokines including interlukin‐6 (IL‐6) and tumor necrosis factor‐α (TNFα) in colon, blood and liver were detected. Lipopolysaccharide (LPS) in colon and blood was detected.

After detection, the multioimc data analysis was performed by Q. Wang *et al*. The biochemical indexes were analyzed by B. Li *et al*.

### Outcome measures

#### ELISA detection and histological examination

Mouse ELISA kits were purchased from Abcam (Cambridge, UK) to detect the levels of ALT, AST, TC, TG, and FFA in blood and liver. Concentrations of IL‐6 and TNFα in the colon, blood, and liver, as well as LPS level in colon and blood were examined by corresponding ELISA kits. Hematoxylin and eosin (H&E) staining was performed on tissue slices of the heart, liver, spleen, lungs, kidneys, and stomach to study organ damage. Periodic acid‐Schiff (PAS) staining was used detect the presence of specific sugars and carbohydrates in liver. Oil Red O (lipid) staining was used to stain lipid and adipocytes in liver.

#### Shotgun metagenomic sequencing and analysis

##### DNA extraction

Fecal samples were thawed on ice for DNA extraction using the QIAamp DNA Stool Mini Kit (Qiagen, Hilden, Germany), and the extracts were treated with DNase‐free RNases (Qiagen) to eliminate RNA contamination according to the manufacturer's instructions. The quantity and quality of DNA were determined by using a NanoDrop spectrophotometer, Qubit Fluorometer (Quant‐iTTM dsDNA BR Assay Kit) and 1.5% agarose gel electrophoresis, respectively. The test results showed all samples meet the requirements for library construction.

##### DNA library construction, sequencing, and filtering

A DNA library was constructed as previously described [24]. Paired‐end (PE) library with an insert size of 350 bp was constructed for each sample, followed by high‐throughput sequencing with PE reads of 2 × 150 bp in length using BGI‐SEQ500 platform (BGI‐Shenzhen, Shenzhen, China).

High‐quality reads were obtained by filtering low‐quality reads with ambiguous “N” bases and adapters (fastp, with parameters: ‐Q, ‐‐thread = 16, ‐‐length_required = 50, ‐‐n_base_limit = 2, ‐‐compression = 6). Host DNA contamination was removed by mapping to GRCm39‐mm39 genome reference consortium by bowtie2 (bowtie2, with parameters: ‐p 48, ‐x mm39_index, ‐‐un‐conc‐gz *RH.fq.gz, ‐S/dev/null). Over 90% of the raw reads were remained to be clean reads, and an average of 10 gigabytes of clean data were obtained for each sample.

##### Taxonomic and functional profiling acquisition

Taxonomic profiles were generated from clean reads using Metagenomic Phylogenetic Analysis (Metaphlan) 3.0 (‐ input_type fastq ‐ ignore_viruses ‐ nproc 6) as described [25]. metaphlan is a computational tool for profiling the composition of microbial communities from metagenomic shotgun sequencing data, which relies on unique clade‐specific marker genes identified from ~ 17 000 reference genomes (~ 13 500 bacterial and archaeal, ~ 3500 viral, and ~ 110 eukaryotic).

For functional profiling, HUMAnN 3.0 (‐i input_clean_data ‐o output ‐‐threads 10 ‐‐memory‐use maximum ‐‐remove‐temp‐output) was used to efficiently and accurately profile the abundance of microbial metabolic pathways and other molecular functions from the metagenomic sequencing data (clean data) according to the reference [25].

##### Diversity calculation

Alpha diversity (r 4.0.3 vegan: diversity (data, index = ‘richness/Shannon/Simpson/InSimpson’)) was calculated using the richness, Shannon index, Simpson's index, and Inverse Simpson's index of the taxonomic profiles. Beta diversity (r 4.0.3 ape: pcoa (‘bray_curtis distance’, correction = “none”, rn = NULL) between‐sample diversity, r 4.0.3 vegan: diversity (data, index = ‘bray_curtis distance’)) was calculated using the bray_curtis distance depending on the taxonomic profiles.

Permutational Multivariate Analysis of Variance (PERMANOVA), was performed on Bray–Curtis's distance and 999 permutations in r (r 4.0.3: adonis (dist~phe, permutations = 1000)) to study the influencing factors of taxonomy between two groups.

#### Transcriptomic sequencing and analysis

##### RNA extraction and library construction

Total RNA from the colonic and liver tissues was isolated using TRIzol Reagent (Thermo Fisher Scientific, Carlsbad, CA, USA). The concentration, quality, and integrity of RNA were determined using a NanoDrop spectrophotometer (Thermo Fisher Scientific). Only samples with an RNA integrity number (RIN) ≥ 7.0 were used to generate transcriptomic libraries.

The enriched mRNA was used for transcriptomic library construction using the TruSeq RNA Library Prep Kit v2 (Illumina, San Diego, CA, USA). The library was then sequenced on a NovaSeq 6000 Illumina platform to obtain image files to generate raw data (FASTQ format).

##### Quality control

Filtered reads were obtained by filtering low‐quality reads with ambiguous “N” bases and adapters (fastp, with parameters:‐Q, ‐‐thread = 16, ‐‐length_required = 50, ‐‐n_base_limit = 2, ‐‐compression = 6).

##### Reads mapping

The reference genome and gene annotation files were downloaded from the website (https://hgdownload.soe.ucsc.edu/goldenPath/mm39/bigZips/mm39.fa.gz). The filtered reads were mapped to the reference genome using hisat2 v2.0.5 (hisat2 (daehwankimlab.github.io)).

##### Differential expression analysis

We used HTSeq (0.9.1) statistics to compare the read count values of each gene with their respective original expression levels. Subsequently, we used fragments per kilobase million (FPKM) to standardize gene expression. Differences in gene expression were analyzed using DESeq (version 1.30.0) with the following screening conditions: expression difference multiple |log_2_ (Fold Change) | > 1, significant *P*‐value < 0.05. Simultaneously, we used the R language pheatmap (1.0.8) software package to perform a bidirectional clustering analysis of all different genes in the samples. We organized the heatmap according to the expression levels of a gene across various samples and the expression patterns of different genes in the same sample using the Euclidean method to calculate the distance and the Complete Linkage method for clustering.

##### GO annotation and KEGG enrichment analysis

We mapped all the genes to terms in the GO database and calculated the number of DEGs for each term. Using the top GO terms, we performed GO enrichment analysis on the DEGs. The *P*‐value was calculated using the hypergeometric distribution method (*P*‐value < 0.05 was considered significant enrichment), and the GO terms associated with significant DEGs were used to determine the main functions of the DEGs.

For the KEGG pathway analysis, clusterprofiler (3.4.4) software was used to conduct KEGG pathway enrichment analysis of the DEGs, and significantly enriched pathways with *P* < 0.05 were determined.

#### Metabolomic detection and analysis

##### Sample preparation and extraction

Biological samples were freeze‐dried using a vacuum freeze‐dryer (Scientz‐100F). The freeze‐dried samples were crushed in a mixer mill (MM 400; Retsch, Haan, Nordrhein‐Westfalen, Germany) with zirconia bead for 1.5 min at 30 Hz. The lyophilized powder (50 mg) was mixed in 1.2 mL of 70% methanol solution and vortexed six times for 30 s every 30 min. After centrifugation at 16114 ***g*** for 3 min, the extracts were filtered (SCAA‐104, 0.22 μm pore size; ANPEL, Shanghai, China) and then subjected to ultra‐performance liquid chromatography–tandem mass spectrometry (UPLC‐MS/MS).

##### UPLC conditions

The sample extracts were analyzed using a UPLC‐ESI‐MS/MS system (UPLC, SHIMADZU Nexera X2; MS; Applied Biosystems 6500 Q TRAP (Sciex, Framingham, MA, USA)). The analytical conditions were as follows. UPLC: column, Agilent SB‐C18 (1.8 μm, 2.1 mm × 100 mm). The mobile phase consisted of solvent A: pure water with 0.1% formic acid and solvent B: acetonitrile with 0.1% formic acid. Sample measurements followed a gradient program starting with 95% A and 5% B. Within 9 min, a linear gradient transition of 5% A and 95% B was programed, and was maintained for 1 min. Subsequently, the composition was adjusted to 95% A and 5.0% B within 1.1 min and maintained for 2.9 min. The flow velocity was set to 0.35 mL·min^−1^; The column oven temperature was set to 40 °C, and the injection volume was 2 μL. The effluent was alternately connected to an electrospray ionization triple quadrupole linear ion trap (QTRAP)‐MS system.

##### ESI‐Q TRAP‐MS/MS

The electrospray ionization (ESI) source operation parameters were as follows: source temperature, 500 °C; ion spray voltage (IS), 5500 V (positive ion mode)/−4500 V (negative ion mode); ion source gas I (GSI), gas II (GSII), and curtain gas (CUR) set at 50, 60, and 25 psi, respectively; high collision‐activated dissociation (CAD). Instrument tuning and mass calibration were performed using 10 and 100 μmol·L^−1^ polypropylene glycol solutions in the QQQ and LIT modes, respectively. QQQ scans were acquired in the MRM experiments using a collision gas (nitrogen) set in the medium. The declustering potential (DP) and collision energy (CE) for individual MRM transitions were determined by DP and CE optimization. A specific set of MRM transitions was monitored for each period according to the metabolites eluted within this period.

##### Principal coordinate analysis

Principal coordinate analysis (PCoA) was performed using the ape comp statistical function in r (www.r‐project.org).

##### Differential metabolites selected

Significantly differentially regulated metabolites between HFD‐fed and control mice were determined using the Wilcoxon rank‐sum test (*P* < 0.05).

##### KEGG annotation and enrichment analysis

Identified metabolites were annotated using the KEGG compound database (http://www.kegg.jp/kegg/compound/) and annotated metabolites were mapped to the KEGG Pathway database (http://www.kegg.jp/kegg/pathway.html).

### Statistical methods

Student's unpaired *t*‐test or two‐sample *t*‐test is used to compare the biochemical indexes from two groups. The Wilcoxon rank‐sum test was used for differential analysis of multiomic data. Spearman's rank correlation was used to evaluate the relationship between the altered compounds and the altered gut microbiota, as well as the similarities between samples. The coefficient of association (rho) results is shown as heat maps, and significance is indicated by *P*‐value (+, *P* < 0.05; *, *P* < 0.01).

## Results

### Changes in obesity‐related phenotypes and indexes in HFD‐induced obesity

After 8 weeks of rapid growth period, the body weight (BW) of the HFD‐fed mice has reached an equilibrium stage and slowly increased every week from the ninth week to the end of the experiment (14th week). Although, the BW of the HFD‐fed mice was significantly heavier compared to the control mice from the third week (Fig. S1, Table S1A, *P* < 0.05). Levels of ALT, TC, and TG in the liver were remarkably increased (*P* < 0.05) in HFD‐fed group, meanwhile, the contents of AST and FFA in liver were also higher in HFD‐fed mice (Fig. S2). In the blood, the levels of AST and TC significantly increased (*P* < 0.05), and that of ALT and FFA also increased (Fig. S2). In addition, the concentrations of LPS, IL‐6, and TNFα in the liver, blood, and colon were increased to different extent in HFD‐fed mice (Fig. S3). H&E staining showed no significant damage of the organs caused by HFD feeding (Fig. S4). These results revealed significant changes of the obesity‐related phenotypes and indexes in long‐term HFD‐induced obesity in mice.

### Alterations of the gut microbiota and derived metabolites in long‐term HFD‐induced obesity

**Fig. 1:**
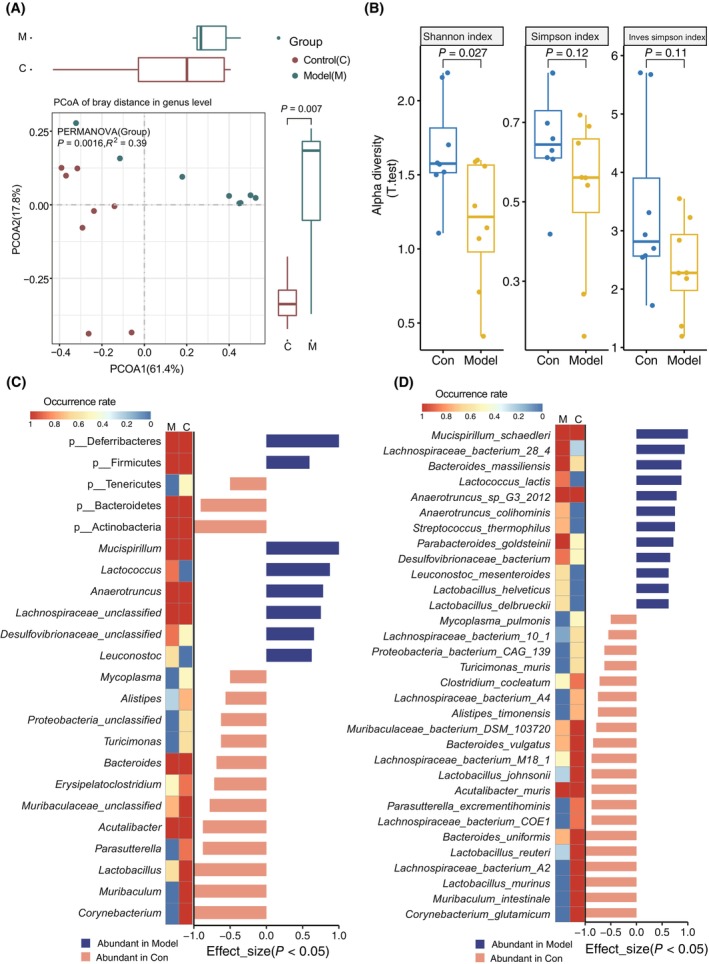
Changes in the gut microbiota composition in mice fed with and without HFD. (A) PCoA based on Bray–Curtis's distance showing significant differences in the gut microbial composition between two groups at genus level. (B) Alpha diversity calculated on Shannon, Simpson, and Inverse Simpson indexes showed a significant decrease of the gut microbial genera in HFD‐fed mice. Significantly different phyla and genera (C) and species (D) between two groups. The color key in C and D suggested the occurrence rate of each phylum, genera and species in HFD‐fed and control group. The columns in C and D represent the effect size of the corresponding species and the color of columns (orange and blue) represent the gut microbial enrichment between two groups. *P* < 0.05 was considered of significance.

Taxonomic profiling was observed by HUMAnN 3.0 (Table S1B). We have focused on differences at phyla (Table S1C), genus (Table S1D) and species (Table S1E) levels. PCoA analysis showed significant difference of the genera (Fig. 1A) and species (Fig. S5A) between control and HFD‐induced HFD‐fed mice. HFD‐fed mice showed reduced gut microbial richness and diversity at both genus (Fig. 1B, Table S1F) and species levels (Fig. S5B, Table S1F). We have explored the characteristics of the high‐abundant species and differential taxonomy between two groups. The most abundant phyla, genera, and species in HFD‐fed mice were explored (Fig. S5C–E). Among the top 20 most abundant genera, 10 were significantly different from those in control mice; of these, *Anaerotruncus* and *Mucispirillum* were significantly abundant, whereas *Erysipelatoclostridium*, *Parabacteroides*, *Corynebacterium*, *Bacteroides*, *Muribaculum*, and *Lactobacillus* were significantly decreased in HFD‐fed mice (Fig. S5D). Compared with the controls, 16 significantly different species in HFD‐fed mice were identified from the top 20 most abundant species; in which *Anaerotruncus* sp. G3‐2012, *Lachnospiraceae* bacterium 28‐4, and *Mucispirillum schaedleri* were significantly abundant, while *Lactobacillus johnsonii*, *Lactobacillus reuteri*, *Bacteroides vulgatus*, *Bacteroides uniformis*, *Lachnospiraceae* bacterium COE1, *Corynebacterium glutamicum*, *Muribaculaceae* bacterium DSM 103720, *Lachnospiraceae* bacterium A2, *Muribaculum intestinale*, and *Lactobacillus murinus* were reduced in HFD‐fed mice (Fig. S5E).

For the differential species, the RA of the phyla Bacteroidetes, Actinobacteria, and Tenericutes was noticeably increased, whereas that of Firmicutes and Deferribacteres was significantly reduced in HFD‐fed mice (Fig. 1C, Fig. S5C). The RA of the genera *Mucispirillum*, *Lactococcus*, *Anaerotruncus*, and *Leuconostoc* was significantly increased, while that of *Mycoplasm*a, *Alistipes*, *Proteobacteria*, *Turicimonas*, *Bacteroides*, *Erysipelatoclostridium*, *Acutalibacter*, *Parasutterella*, *Lactobacillus*, *Muribaculum*, *Corynebacterium* was significantly decreased in HFD‐fed mice (Fig. 1C). At the species level, the RA of *Lachnospiraceae* bacterium 28‐4, *Anaerotruncus* sp. G3‐2012, *Mucispirillum schaedleri*, *Desulfovibrionaceae bacterium*, *Bacteroides massiliensis*, *Parabacteroides goldsteinii*, *Leuconostoc mesenteroides*, *Anaerotruncus colihominis*, *Lactobacillus delbrueckii*, *Lactobacillus helveticus*, *Lactococcus lactis*, and *Streptococcus thermophilus* were increased in HFD‐fed mice, while that of *Lactobacillus murinus*, *Muribaculum intestinale*, *Lachnospiraceae* bacterium A2, *Muribaculaceae* bacterium DSM 103720, *Corynebacterium glutamicum*, *Lachnospiraceae* bacterium COE1, *Bacteroides uniformis*, *Lactobacillus johnsonii*, *Lactobacillus reuteri*, *Bacteroides vulgatus*, *Alistipes timonensis*, *Turicimonas muris*, *Lachnospiraceae* bacterium M18‐1, *Proteobacteria* bacterium CAG 139, *Acutalibacter muris*, *Clostridium cocleatum*, *Lachnospiraceae* bacterium 10‐1, *Parasutterella excrementihominis*, *Lachnospiraceae* bacterium A4 remarkably decreased (Fig. 1D).

HUMAnN 3.0 was used to efficiently and accurately profile the presence/absence and enrichment of microbial pathways from metagenomic sequencing data (Table S1G). We identified 59 significantly pathways according to the effect size (*P* < 0.05). Superpathways of glycolysis, pyruvate dehydrogenase, tricarboxylic acid cycle, glyoxylate bypass, glucose, xylose, and sucrose degradation were significantly enriched in controls, while pathways related to deoxyribonucleotide *de novo* biosynthesis and degradation, and amino acid biosynthesis were significantly enriched in HFD‐fed mice (Fig. S6). Further, we identified the pathways modulated by *Lactobacillus* spp., suggested beneficial bacteria, which were significantly decreased in the HFD‐fed mice. *Lactobacillus reuteri* correlated with d‐galactose degradation I, coenzyme A biosynthesis I, adenine and adenosine salvage II, and purine ribonucleoside degradation. *Lactobacillus johnsonii* was involved in the superpathway of coenzyme A biosynthesis III. *Lactobacillus murinus* was mainly associated with lactose and galactose degradation I, coenzyme A biosynthesis I, adenosine/guanosine ribonucleotide *de novo* biosynthesis, purine ribonucleoside degradation, stachyose degradation, inosine‐5‐phosphate biosynthesis, l‐lysine biosynthesis, peptidoglycan biosynthesis, UMP biosynthesis, and UDP‐*N*‐acetylmuramoyl‐pentapeptide biosynthesis, all of which were significantly reduced in the gut of HFD‐fed mice. These results suggesting *Lactobacillus* spp. and their involved functional pathways were decreased in HFD‐fed mice.

**Fig. 2:**
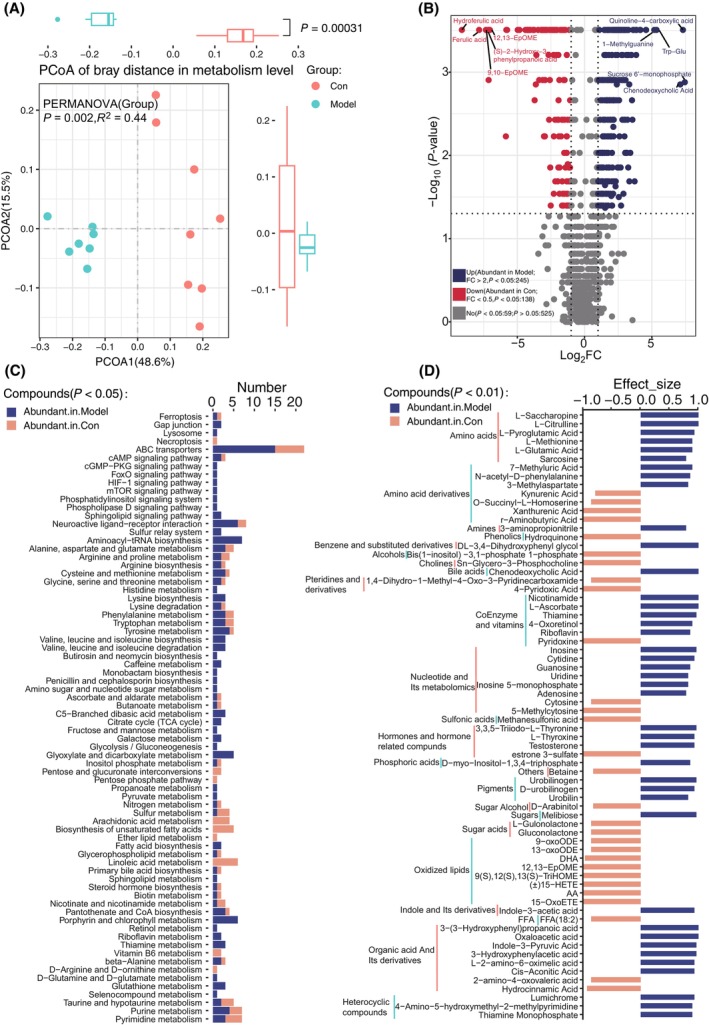
Differential analysis of colon metabolic signatures of HFD‐fed and control mice. (A) PCoA based on Bray–Curtis's distance showed significant difference in the colonic metabolites between two groups of mice. (B) Volcano plot showed the differences in colonic metabolites between two groups. Red and blue dots represent the significantly altered colonic metabolites in control and HFD‐fed mice, respectively (*P* < 0.05). (C) KEGG pathways involved for the significantly different colonic metabolites (*P* < 0.05). The columns represent the number of metabolites. (D) Significantly different colonic metabolites between two groups (*P* < 0.01). The columns represent the effect size of metabolites. The color of the columns in C and D represents enrichment of colonic metabolites between two groups.

To further investigate the functional changes of the gut microbiota, we have examined the gut microbiota‐derived metabolites (Table S1H). PCoA revealed that the metabolites were significantly different between the control and HFD‐fed mice (Fig. 2A, *P* = 0.00031). Compared with the control mice, of the 383 significantly different metabolites, 245 were abundant, while 138 were less abundant in HFD‐fed mice than in control mice (Fig. 2B). Interestingly, amino acids and their derivatives, coenzymes and vitamins, nucleotides and their metabolites, hormones and hormone‐related compounds, and organic acids and derivatives were increased in HFD‐fed mice, while saccharides, oxidized lipids, pteridines, and derivatives were increased in controls (Fig. 2D). These results suggested that the colon of HFD‐fed mice might have a stronger ability to absorb lipids. KEGG pathway analysis revealed that the main pathways enriched in HFD‐fed mice were involved in amino acid biosynthesis, degradation, and metabolism, polysaccharide metabolism, the citrate cycle, glycolysis/gluconeogenesis, and fatty acid biosynthesis. However, pathways involved in lipid metabolism, unsaturated fatty acid biosynthesis, and the pentose phosphate pathway were enriched in the controls (Fig. 2C, Table S1I).

### Gut microbiota and metabolites modulate colonic gene expression

**Fig. 3:**
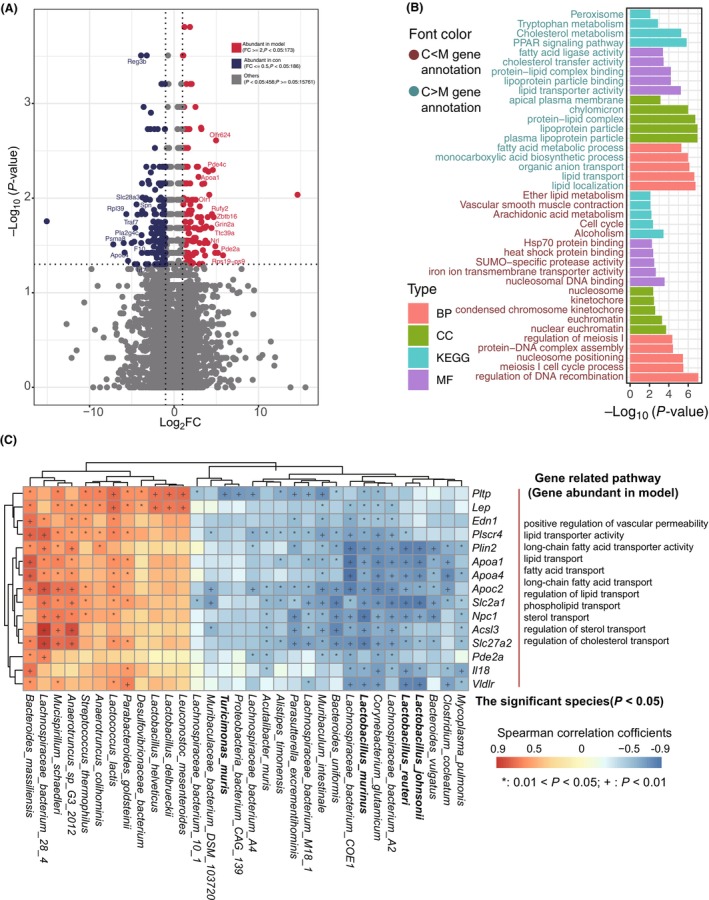
Colonic gene expression in HFD‐fed and control mice. (A) PCoA showing significant differences in the colonic gene expression of the two groups of mice (*P* < 0.05). Red and blue dots represent the genes highly abundant in HFD‐fed and control mice, respectively. (B) GO annotation and KEGG pathway analysis of the significant colonic DEGs between two groups (*P* < 0.05). Colors of the bars meant different levels of GO including CC, MF and BP, and KEGG. The color of the text distinguishes the two groups. (C) Correlation analysis between the significantly different gut microbiota and colonic DEGs involved in lipid absorption, transport, and metabolism. Red and blue squares meant positive and negative correlations, respectively. *P < 0.05; ^+^ *P* < 0.01.

To elucidate the effect of the gut microbiome on host gene expression and function, colonic gene expression between HFD‐fed and control mice were examined. A total of 359 DEGs were identified in the colon between two groups, of which 173 DEGs were significantly upregulated while 186 DEGs were downregulated in HFD‐induced obese mice (Fig. 3A, Table S1K). GO annotation revealed that the upregulated DEGs were mainly related to lipoprotein particles, plasma lipoprotein particles, protein‐lipid complexes, chylomicrons, and apical plasma membranes as cellular components (CC), fatty acid ligase activity, cholesterol transfer activity, lipoprotein particle binding, and lipid transport activity as molecular functions (MF), and fatty acid metabolic processes, lipid transport, and localization as biological processes (BP). KEGG analysis showed that the upregulated DEGs were mainly involved in the peroxisome, tryptophan metabolism, cholesterol metabolism, and PPAR signaling pathways, suggesting that colonic genes related to lipid metabolism and transport were significantly overexpressed in HFD‐fed mice (Fig. 3B, Table S1L). In contrast, the downregulated DEGs were mainly related to the nucleosome, kinetochore, condensed chromosome kinetochore, euchromatin, and nuclear euchromatin as CC, hsp70 protein binding, heat shock protein binding, and SUMO‐specific protease activity as MF, and the regulation of meiosis I, protein‐DNA complex assembly, nucleosome positioning, and meiosis I cell cycle process as BP. KEGG pathway analysis showed that the downregulated DEGs were mainly involved in pathways such as lipid metabolism, vascular smooth muscle contraction, arachidonic acid metabolism, the cell cycle, and alcoholism (Fig. 3B, Table S1L).

Correlation analysis was performed to investigate the relationships between the significantly different bacteria, microbial metabolites, and colonic DEGs in HFD‐induced obese mice (Table S1J). Bacterial species enriched in HFD‐induced obese mice, namely, *Mucispirillum schaedleri*, *Lactococcus lactis*, Lachnospiraceae bacterium 28‐4, *Bacteroides massiliensis*, *Streptococcus thermophilus*, *Anaerotruncus colihominis*, *Anaerotruncus* sp. G3:2012, *Lactobacillus delbrueckii*, *Lactobacillus helveticus*, *Leuconostoc mesenteroides*, and *Parabacteroides goldsteinii* were significantly and positively associated with the metabolites increased in and DEGs upregulated in the colon of HFD‐fed mice (Fig. 3C, Fig. S7, Table S1M). These results suggested an association between the gut microbiota, colonic gene expression, and microbial metabolites, wherein HFD‐induced obese mice exhibit increased lipid biogenesis, whereas the controls exhibit increased lipid catabolism.

### Associations between the gut microbiota‐derived metabolites and blood metabolism in HFD‐fed mice

The plasma metabolites were investigated between two groups (Fig. S8A, Table S1N). Fifty‐one metabolites, including amino acids, carnitine, choline, FFA, hormones and hormone‐related compounds, LPA, LPC, oxidized lipids, sphingomyelin (SM), and saccharides, were significantly increased in the plasma of HFD‐fed mice, whereas 39 metabolites, including coenzymes, vitamins, organic acids, and their derivatives, were significantly increased in the plasma of control mice (Fig. S8B,D). KEGG analysis revealed that the HFD‐fed mice‐enriched plasma metabolites were mainly focused on pathways such as ABC transporters, amino acid metabolism, fructose and mannose metabolism, galactose metabolism, pentose and glucuronate interconversion, propanoate metabolism, starch and sucrose metabolism, unsaturated fatty acid biosynthesis, fatty acid biosynthesis, elongation and degradation, and glycerolipid and glycerophospholipid metabolism. On the contrary, the control‐enriched metabolites were primarily concentrated on the AMPK signaling pathway, cysteine and methionine metabolism, glycine, serine, and threonine metabolism, tryptophan metabolism, valine, leucine, and isoleucine biosynthesis, citrate cycle, glycolysis/gluconeogenesis, pentose phosphate pathway, pyruvate metabolism, pantothenate and CoA biosynthesis, and purine metabolism (Fig. S8C, Table S1O).

Then we have explored the relationship between blood metabolites and gut microbiota and derived metabolites (Table S1P). Ninety‐one common metabolites were observed between blood and feces (Fig. S9). In which, 45 metabolites including *S*‐(Methyl) glutathione, d‐Proline betaine, Kynurenic Acid, l‐Methionine, 3‐Chloroaniline, l‐Carnitine, Proline betaine, Allantoin, Uric acid, LPC, LPE and etc. showed the same enrichment, saying both high or low in feces and blood of HFD‐fed mice. The other 46 metabolites including EPA, DHA, FFA, 5‐Methyluridine and etc. showed contrary results between blood and feces in HFD‐fed mice. Correlation analysis between the 46 metabolites and significant different gut bacteria between two groups were explored (Fig. S10). Interestingly, the HFD‐fed mice‐enriched species were significantly positively correlated with HFD‐fed mice‐enriched colonic metabolites, but were obviously negatively correlated with HFD‐fed mice reduced colonic metabolites. For the control mice‐enriched species, they were significantly positively correlated with control mice‐enriched fecal metabolites but negatively correlated with the plasma‐enriched metabolites in control mice. These results suggested that the complex relationship between gut microbiota, gut microbiota‐derived metabolites and blood metabolites.

### Liver gene expression and metabolites in HFD‐fed mice

The gut‐liver axis plays an important role in host metabolism and it is important to study liver gene expression and metabolites, as well as their associations with the gut microbiota and metabolites.

**Fig. 4:**
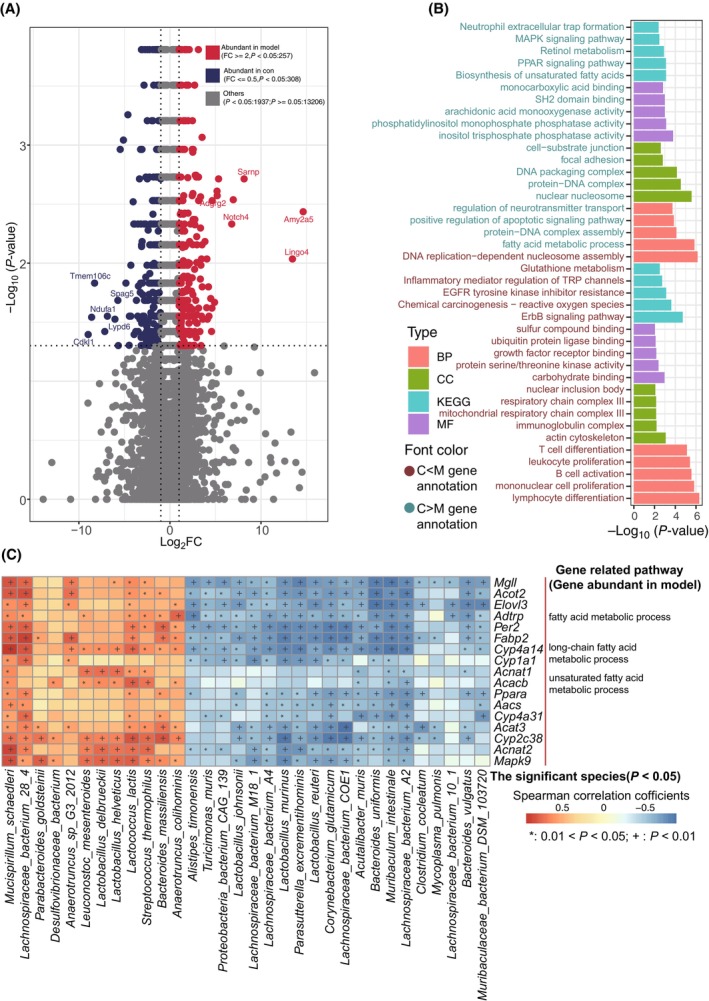
Differences in liver gene expression between HFD‐fed and control mice. (A) Volcano plot showed significantly different liver genes between two groups. (B) GO annotation and KEGG pathway analysis of the significant liver DEGs between two groups (*P* < 0.05). The color of bars suggested the different levels of GO (CC, MF and BP) and KEGG, the color of the axis text suggested two different groups. (C) Association analysis between the significantly different gut microbiota and the DEGs that involved in lipid metabolism in the HFD‐fed mice. Red and blue squares meant positive and negative correlations, respectively. *P < 0.05; ^+^ *P* < 0.01.

RNA‐seq revealed 565 liver DEGs that were significantly different between HFD‐fed and control mice (Table S1Q). Of these, 257 DEGs were significantly upregulated and 308 were downregulated in HFD‐fed mice (Fig. 4A). GO annotation and KEGG pathway analyses of these DEGs (Fig. 4B, Table S1R) revealed that the upregulated DEGs were mainly associated with nuclear inclusion bodies, respiratory chain complex II, mitochondrial respiratory chain complex III, immunoglobulin complex, and the actin cytoskeleton, whereas the downregulated DEGs were mainly associated with cell‐substrate junctions, focal adhesions, DNA packaging complexes, protein‐DNA complexes, and nuclear nucleosomes as CC. Further, the upregulated DEGs were mainly associated with sulfur compound binding, ubiquitin protein ligase binding, growth factor receptor binding, protein serine/threonine kinase activity, and carbohydrate binding, while the downregulated DEGs with inositol trisphosphate phosphatase activity, phosphatidylinositol monophosphate phosphatase activity, and arachidonic acid monooxygenase activity as MF. Finally, the upregulated DEGs were mainly associated with T cell differentiation, leukocyte proliferation, B cell activation, mononuclear cell proliferation, and lymphocyte differentiation, whereas downregulated DEGs with DNA replication‐dependent nucleosome assembly, fatty acid metabolism, protein‐DNA complex assembly, and regulation of neurotransmitter transport as BP. KEGG pathway analysis showed that glutathione metabolism, inflammatory mediator regulation of TRP channels, EGFR tyrosine kinase inhibitor resistance, chemical carcinogenesis‐reactive oxygen species, and the ErbB signaling pathway were the main pathways associated with DEGs upregulated in HFD‐fed mice. Whereas neutrophil extracellular trap formation, the MAPK signaling pathway, retinol metabolism, PPAR signaling pathway, and unsaturated fatty acid biosynthesis were the main pathways associated with DEGs upregulated in control mice. Correlation analysis showed that the upregulated DEGs in HFD‐fed mice were positively correlated with bacterial species enriched in HFD‐fed mice and negatively correlated with those enriched in control mice (Fig. 4C), indicating that the gut microbiota regulates liver gene expression, and vice versa.

Principal coordinate analysis of the liver metabolites showed a marked difference between the two groups (Fig. S11A, Table S1S). A total of 69 significantly different metabolites were identified, of which 13 were increased in HFD‐fed mice and 56 were increased in the controls (Fig. S11B). Oxidized lipids, organic acids and their derivatives, coenzymes and vitamins, pteridines, and their derivatives were significantly increased in HFD‐fed mice, while carbohydrates were increased in the controls (Fig. S11D). The KEGG pathways associated with these metabolites are shown in Fig. S11C. Interestingly, the AMPK, HIF‐1, mTOR, and PI3K‐Akt signaling pathways, fructose, mannose, galactose, glucose, propanoate, pyruvate, starch and sucrose, and nitrogen metabolism, unsaturated fatty acid biosynthesis, ether lipid metabolism, fatty acid biosynthesis, fatty acid degradation, fatty acid elongation, glycerophospholipid metabolism, and sphingolipid metabolism were significantly enriched in HFD‐fed mice. ABC transporters, neuroactive ligand‐receptor interactions, amino acid metabolism, tryptophan metabolism, oxidative phosphorylation, arachidonic acid metabolism, primary bile acid biosynthesis, and steroid hormone biosynthesis were enriched in controls. These results suggested that the livers of HFD‐fed mice exhibited increased carbohydrate and lipid metabolism as well as fatty acid biosynthesis, while the controls exhibited increased amino acid, sterol, steroid, and vitamin metabolism (Table S1T).

**Fig. 5:**
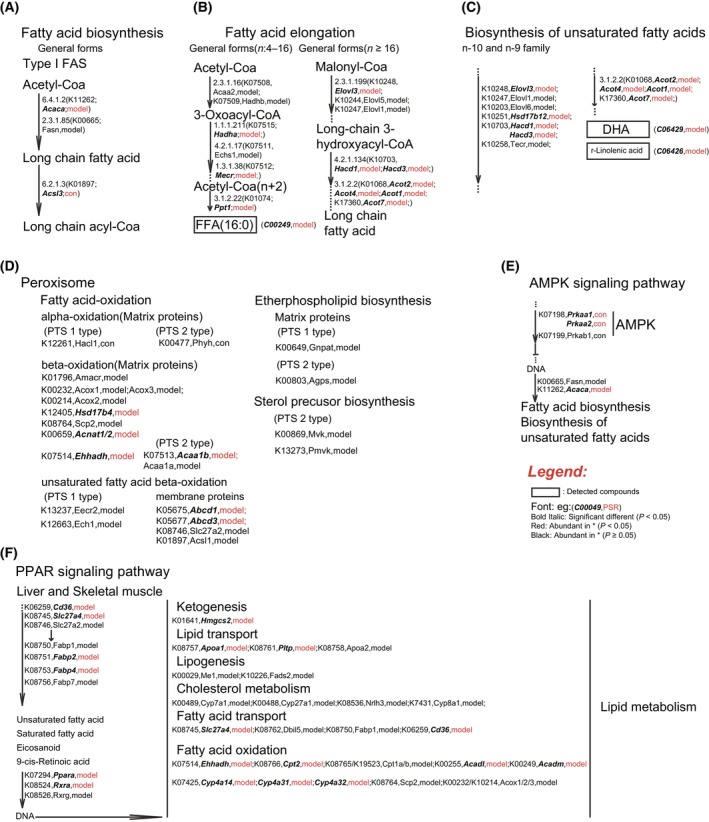
Seven KEGG pathways involved in liver lipid metabolism by liver differential genes between two groups. (A) Fatty acid biosynthesis. (B) Fatty acid elongation; (C) Biosynthesis of unsaturated fatty acids; (D) Four KEGG pathways involved by Peroxisome; (E) AMPK signaling pathway; (F) PPAR signaling pathway. The front and back of the arrows are compounds, next to the arrows are the genes involved in this step. The compounds in the rectangular circle represent those metabolites detected by untargeted metabolomics. Bold Italic font in bracket: Significantly different DEGs between two groups (*P* < 0.05); Red font in bracket: Significantly abundant in related group (*P* < 0.05); Black font in bracket: Higher in related group (*P* ≥ 0.05).

**Fig. 6:**
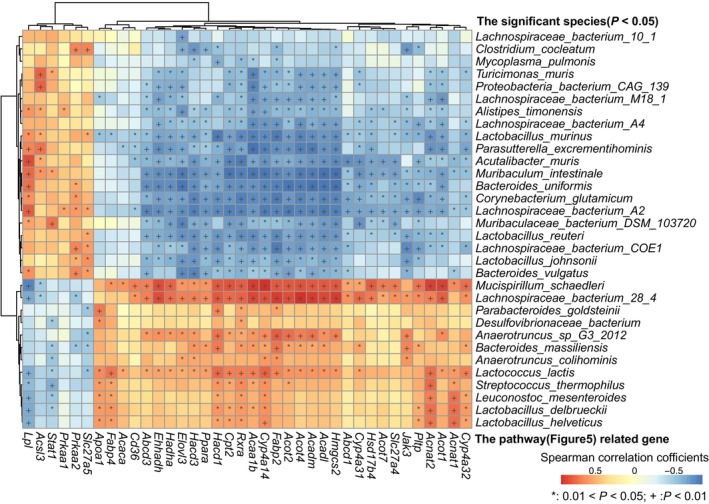
Heatmap showed the correlation analysis between the significantly different microbial species and the significantly different liver genes involved in lipid metabolism. Red and blue squares meant positive and negative correlations, respectively. *P < 0.05; ^+^ *P* < 0.01.

To further explore the effects of the gut microbiome on hepatic lipid metabolism, we selected differential genes related to lipid metabolism, including fatty acid biosynthesis, fatty acid elongation, unsaturated fatty acid biosynthesis, peroxisome, AMPK signaling pathway, and the PPAR signaling pathway (Fig. 5). Interestingly, most differential genes involved in these pathways were highly expressed in HFD‐induced obese mice. Spearman's rank correlation analysis was used to explore the relationships between significantly different species and significantly altered DEGs related to lipid metabolism (Fig. 6, Table S1U). DEGs including *Slc27a5*, *Prkaa2*, *Prkaa1*, *Stat1*, *Acsl3*, and *Lpl* were enriched in the livers of controls and were positively correlated with bacteria abundant in control mice such as *Turicimonas muris*, *Alistipes timonensis*, *Lactobacillus murinus*, *Parasutterella excrementihominis*, *Acutalibacter muris*, *Muribaculum intestinale*, *Bacteroides uniformis*, *Lactobacillus johnsonii*, and *Bacteroides vulgatus*. In contrast, DEGs enriched in HFD‐fed mice, including *Fabp4*, *Apoa1*, *Cyp4a14*, *Acaa1b*, *Rxra*, *Cpt2*, *Hacd1*, *Cyp4a32*, *Acnat1*, *Acot1*, *Acnat2*, *Pltp*, and *Jak3* were significantly and positively associated with obesity‐abundant bacteria, such as *Mucispirillum schaedleri*, *Lachnospiraceae* bacterium 28‐4, *Anaerotruncus* sp. G3:2012, *Lactococcus lactis*, *Leuconostoc mesenteroides*, *Lactobacillus delbrueckii* and *Lactobacillus helveticus*. These results indicated close associations between gut microbiota and liver gene expression.

## Discussion

Metagenomics, metabolomics, and transcriptomics in HFD‐induced obesity animal model and obese human individuals have been studied alone or jointly, however, changes and relationships among these multiomics in the same individuals remain unclear. Therefore, our study aimed at investigating the characteristics and changes of the gut microbiota and microbial metabolites, colonic and liver gene expression, blood and liver metabolites, as well as the associations among these multiomics between HFD‐fed mice and healthy controls, which would provide theoretical and data support for future obesity‐related studies.

The gut microbiota exerts direct effects on the digestion, absorption and metabolism of food. Evidences have showed that microbial imbalance in the gut contributes to obesity [26, 27]. Lower alpha diversity and higher BMI was showed to be most consistent in obese populations [28]. In animal studies, however, studies on the effects of high‐fat diets on the host have drawn inconsistent results. It was revealed by 16S rRNA gene sequencing that the HFD increased gut microbial diversity, enriched Desulfovibrionaceae and *Mucispirillum*, meanwhile, reduced the abundance of *Lactobacillus*, *Bifidobacterium*, *Akkermansia*, *Faecalibaculum*, and *Blautia* [6]. However, in another study, Liu *et al*. [29] revealed decreased microbial diversity and differential metabolite profiles in HFD‐fed mice by 16S rRNA sequencing and metabolomics analysis of fecal samples. In our study, we revealed that the gut microbial diversity and richness were significantly reduced in HFD‐fed mice by shotgun metagenomic sequencing. Phyla Bacteroidetes were significantly reduced while Firmicutes were significantly abundant in HFD‐fed mice, a greater Firmicutes/Bacteroidetes (F/B) ratio was observed for HFD‐fed mice, which was consistent with existing research [30]. For genera, the RA of Desulfovibrionaceae, *Mucispirillum*, *Mucispirillum schaedleri* was enriched while that of *Lactobacillus*, *Lactobacillus murinus*, *Lactobacillus reuteri*, *Lactobacillus johnsonii*, *Bacteroides*, *Bacteroides uniformis*, *Bacteroides vulgatus* was reduced in HFD‐fed mice, which was consistent with previous studies [6]. *Lactococcus chungangensis CAU 28* was reported to alleviate diet‐induced obesity and adipose tissue metabolism *in vitro* and in mice fed a HFD [31], we found that *Lactococcus* and *Lactococcus lactis* were enriched in HFD‐fed mice which might be a feedback regulation. Interestingly, *Lactobacillus helveticus* and *Lactobacillus delbrueckii* were enriched in HFD‐fed mice in our study. Emerging research suggested that *lactobacillus delbrueckii* may play a role in weight management and the prevention of obesity [32]. *Lactobacillus helveticus* is a probiotic with high proteolytic activity, and can efficiently metabolizes lactose and galactose [33]. Additionally, *Anaerotruncus* and Lachnospiraceae were reported to be enriched in the gut of healthy people on a high SFAs diet [34], which was consistent with our results. *Leuconostoc mesenteroides*, mediating an electrogenic pathway to attenuate the accumulation of abdominal fat mass induced by HFD [35], was enriched in HFD‐fed mice in our study. *Streptococcus thermophilus* positively correlated with obesity [36], was enriched in HFD‐fed mice. *Parabacteroides goldsteinii*, represents novel prebiotics and probiotics that may be used to treat obesity and type 2 diabetes [37], was significantly enriched in HFD‐fed mice. *Muribaculum intestinale*, significantly increased in the gut in the inulin group and biomarker of high fructose‐induced intestinal microbiota disorders [38], was reduced in HFD‐fed group in our study. *Corynebacterium*, *Corynebacterium glutamicum* S9114 is commonly used for industrial glutamate production, reduced in HFD‐fed mice in our study, reminding the importance of glutamate in obesity. *Parasutterella*, *Parasutterella excrementihominis*, linking to the fatty acid biosynthesis pathway for body weight gain in response to a carbohydrate‐rich diet in obesity development [39], was reduced in HFD‐fed mice *Alistipes*, *Alistipes timonensis*, as described to be negatively associated with body weight gain [40], was reduced in HFD‐fed mice in our study. In addition, we have also revealed a decrease in *Turicimonas*, *Turicimonas muris*, *Mycoplasma*, *Mycoplasma pulmonis*, *Erysipelatoclostridium*, *Clostridium cocleatum*. Taking together, we have revealed that the previously reported and some novel changes in gut microbiota in HFD‐induced HFD‐fed mice, some of these changes might be a feedback regulation of the gut.

The predicted metabolic pathways indicated that the gut microbiota in HFD‐fed mice was mainly linked to the superpathways of glycol metabolism and degradation, superpathway of glycolysis, pyruvate dehydrogenase, tricarboxylic acid cycle, and glyoxylate bypass, and superpathway of sucrose/glucose and xylose degradation, whereas that of the controls was mainly focused on amino acid biosynthesis and superpathway of guanosine/adenosine nucleotides *de novo* biosynthesis. To further analyze the function of the gut microbiota, we have detected the fecal metabolomics significant differences in the gut microbiota‐derived metabolites were observed. Two hundred and forty‐five metabolites, including amino acids, coenzymes and vitamins, nucleotides and their metabolites, organic acid and its derivatives were increased, while 138 metabolites, including saccharides and oxidized lipids, were significantly reduced in HFD‐fed mice compared with healthy controls. Indole‐3‐acetic acid has been shown to drive the emergence of obesity [41], was enriched in HFD‐induced obesity. In addition, l‐glutamic acid, Indole‐3‐Pyruvic Acid were also enriched in HFD‐induced obesity. Glutamate diet, along with HFD, was thought to play a role in the occurrence of fat deposition and obesity [42].

Gut microbiota could affect the physiology and healthy state of the body by directly function on the colon tissues or go across the gut mucosal barrier to enter into the bloodstream and target organs [43]. So, we have explored the colonic and liver gene expression. Colonic transcriptomic analysis was further analyzed to verify the above results since the gut microbiota and their metabolites might change the gene expression on colon tissues. A total of 173 DEGs participating in fatty acid metabolic process, lipid transport and localization, peroxisome, cholesterol metabolism, tryptophan metabolism, and PPAR signaling pathways were significantly upregulated in HFD‐fed mice. Tryptophan concentrations negatively correlated with adiponectin and were significantly higher in prediabetes and metabolically unhealthy obesity [44]. These results further confirmed that HFD increased the colonic gene expression of lipid transport, localization, and metabolism in mice, which might be one of the causes of body obesity under HFD feeding. Liver is the primary organ for lipid metabolism, so we have subsequently detected gene expression in the liver. Totally 565 DEGs between HFD‐fed mice and controls were observed, of which 257 DEGs significantly upregulated in HFD‐fed mice were mainly involved in fatty acid metabolic process, biosynthesis of unsaturated fatty acids, positive regulation of apoptotic signaling pathway, MAPK signaling pathway, and PPAR signaling pathways and etc. It was reported that activation on the p38‐MAPK signaling pathway could inhibit lipid accumulation to reduce the incidence of obesity [45]. An upregulation of genes participated in MAPK signaling pathway and fatty acids metabolic process in HFD‐fed mice, suggesting an increase in lipid metabolism, which might be a feedback regulation of the liver under HFD intake, and eventually, the liver becomes overloaded and impaired. For the 308 downregulated DEGs in HFD‐fed mice, they were mainly focused on immune cell activation, proliferation and differentiation such as T cell differentiation, leukocyte proliferation, B cell activation, mononuclear cell proliferation, lymphocyte differentiation, and ErbB signaling pathway. Obesity is a state of low‐grade chronic inflammation, during which, signaling via cytokines of the TNF family mediate cell death and inflammation within the adipose tissue, eventually resulting in lipid spill‐over, glucotoxicity and insulin resistance, ultimately lead to ectopic lipid deposition, glucose intolerance and other metabolic complications with life‐threatening consequences [46]. A reduction in genes related on immune cell activity suggesting a decline in immune regulation of the liver under HFD feeding, together with burdened liver function, finally result in liver damage.

Blood metabolite levels are under the influence of environmental and genetic factors [47]. Blood metabolic diversity has potential new opportunities for drug development and disease understanding [48]. So, we detected the blood metabolism in two groups of mice. Plasma metabolites further reinforced higher enrichment of metabolites including amino acids, free fatty acids, hormones, hormone‐related compounds, LPA, LPC, oxidized lipids, and saccharide‐related substances in HFD‐fed mice, however, low concentrations of carbohydrates such as stachyose, lactose, d‐Trehalose, suggesting high levels of small molecules associated with lipid metabolism, which is consistent with high absorption and transportation of lipid‐related substances. Gut‐liver axis has revealed that communications between gut and liver has played a key role in liver diseases [49]. The liver plays a significant role in metabolism, glucose synthesis, and storage [50]. We have examined the liver metabolomics in two groups of mice. The results revealed that amino acids, free fatty acids, hormones, uridine, creatine phosphate, DHA, EPA, Sphingosine 1‐phosphate, and carbohydrates were significantly enriched in HFD‐fed obesity. These results suggested high metabolic ability of liver in mice under HFD feeding.

We have further investigated the common metabolites among colon, blood, and liver by Venn diagram (Fig. S12, Table S1V). Totally, 25 metabolites were observed among colon, blood and liver metabolites. Among which, 65 metabolites including uridine, l‐methionine, kynurenic acid (KA) were increased while uric acid, l‐carnitine, allantoin etc. were reduced in the colon and blood of HFD‐fed mice. Uridine metabolism is tightly relating to glucose homeostasis and lipid and amino acid metabolism [51]. Methionine restriction was reported to affect lipid metabolism and can prevent obesity in mice [52]. An increase in KA in skeletal muscles could increase thermogenesis in the long term and limit weight gain, insulin resistance and inflammation, also KA presents in breast milk and may act as an anti‐obesity agent in infants [53]. Uric acid (UA), a clinical marker of oxidative stress, is a promising biomarker for future weight gain and cardiometabolic risk in young adults. Carnitine supplementation could significantly decrease weight loss [54]. Allantoin has been reported to improve lipid metabolism in HFD‐fed mice [55]. Meanwhile, we have also observed 34 metabolites that were significantly changed in plasma and liver of the HFD‐fed mice. LPC, LPE, FFA and etc. were increased in HFD‐fed mice, while heparin, Trimethylamine *N*‐Oxide (TMAO), hexanoyl glycine, salicylaldehyde, 3‐Indolepropionic Acid (IPA). Heparin is reported to reduce weight in obese humans [56]. TMAO is not only associated with increased risk of diabetes and cardiovascular disease morbidity and mortality, but also has been revealed positive dose‐dependent associations between circulating TMAO concentration and obesity [57]. Lower circulating glycine levels have been consistently observed in T2DM, and NAFLD [58], and obesity is characterized by a decrease in plasma glycine concentration [59]. Salicylaldehyde was reported to be increased under exercise, which could control weight [60]. The level of IPA can predict the occurrence of obesity, and IPA supplementation has been shown to improve blood glucose, increase insulin sensitivity, inhibit liver lipid synthesis and inflammatory factors [61]. In conclusion, metabolites significantly related to obesity and lipid metabolism in colon, plasma, and liver have been changed, suggesting 14 weeks of HFD feeding could significantly increase the weight and alter the metabolic characteristics of the mice, and the mice body could self‐regulate lipid balance under long term of HFD intake.

## Conclusion

In conclusion, our study based on HFD‐fed mice revealed interesting changes and complex associations among the gut microbiota, microbial metabolism, colonic and liver gene expression, plasma and liver metabolic signatures between HFD‐fed mice and healthy controls. Our findings have laid the groundwork for a comprehensive understanding of HFD‐induced obesity, offering insights from multiple dimensions through a multiomic approach.

## Conflict of interest

The authors declare no conflict of interest.

### Peer review

The peer review history for this article is available at https://www.webofscience.com/api/gateway/wos/peer‐review/10.1002/2211‐5463.13788.

## Author contributions

BL and XO were in charge of project conception, animal experiments, sample collection, and funding support. JC was responsible for results visualization, manuscript writing and funding support. XL assisted in animal experiments. ZX assisted in multiomic data analysis. XX was responsible for experimental design, manuscript writing. YY was responsible for experimental design and funding support. QW took charge of project conception, multiomic data analysis, methodology, software and figure visualization. All the authors have read and reviewed the manuscript, and agreed with the publication of the work.

## Supporting information


**Fig. S1.** Changes of the body weight in the high‐fat diet‐fed mice and the control mice during the whole experiment.
**Fig. S2.** Contents of ALT, AST, TC, TG, and FFA in the liver and blood of HFD‐fed mice and controls.
**Fig. S3.** Levels of IL‐6, TNFα, and LPS in the colon, blood, and liver of HFD‐fed mice and controls.
**Fig. S4.** H&E staining of the main organs and morphology of the mice.
**Fig. S5.** Changes of the gut microbiota before and after HFD treatment.
**Fig. S6.** Functional changes of the gut microbiota before and after treated with HFD.
**Fig. S7.** Correlation analysis of the significantly different species and metabolites in Fig. 2D.
**Fig. S8.** Differential analysis of metabolites in the plasma of HFD‐fed and control mice.
**Fig. S9.** Correlation analysis of the colon and plasma metabolites in HFD‐fed mice.
**Fig. S10.** Correlation analysis of the significantly different gut microbial metabolites and common metabolites showed contrary enrichment in colon and plasma between two groups.
**Fig. S11.** Variation analysis of the liver metabolisms.
**Fig. S12.** Venn analysis of metabolites from colonic content, liver and plasma.

**Table S1.** Detailed data used for analysis and all the figures of the manuscript except for metagenomic sequencing data.
**Table S1A.** Changes of the body weight in the high‐fat diet‐fed mice and the control mice during the whole experiment.
**Table S1B.** Taxonomic profiling of the mice in two groups.
**Table S1C.** Phyla analysis of the mice in two groups.
**Table S1D.** Genera analysis of the mice in two groups.
**Table S1E.** Species analysis of the mice in two groups.
**Table S1F.** Diversity calculation of the mice in two groups.
**Table S1G.** HUMAnN 3.0 results of the gut microbiota in the two group of mice.
**Table S1H.** Colon metabolome of the mice in two groups.
**Table S1I.** KEGG pathway annotation of the colon metabolites in the two groups of mice.
**Table S1J.** Correlation analysis between the significantly different species and colon metabolites in the two groups of mice.
**Table S1K.** Gene expression of the colon tissues in the two groups of mice using RNA sequencing.
**Table S1L.** GO annotation and KEGG pathway analysis of the colonic DEGs in the two groups of mice.
**Table S1M.** KEGG pathways related to lipid metabolism in colon in the two groups of mice.
**Table S1N.** Plasma metabolites in the two groups of mice.
**Table S1O.** KEGG pathway annotation of the PLASMA metabolites in two groups of mice.
**Table S1P.** Common metabolites anlaysis between colon and plasma in two groups of mice.
**Table S1Q.** Liver gene expression in the two groups of mice using RNA sequencing.
**Table S1R.** GO annotation and KEGG pathway analysis of the liver DEGs in the two groups of mice.
**Table S1S.** Liver metabolites in the two groups of mice.
**Table S1T.** KEGG pathway annotation of the PLASMA metabolites in two groups of mice.
**Table S1U.** Expression of liver genes related to lipid metabolism.
**Table S1V.** Data of Venn diagram among liver, plasma, and colon metabolites.

## Data Availability

The shotgun metagenomic sequencing data and transcriptomic data that support the findings of this study are openly available at BioProject database in National Library of Medicine (NCBI, https://www.ncbi.nlm.nih.gov/) with the accession number of PRJNA1040160. The authors confirmed that the other data including untargeted metabolomic data that supporting the findings of this study are available in the article and its Supporting Information.

## References

[1] Islam MR , Arthur S , Haynes J , Butts MR , Nepal N and Sundaram U (2022) The role of gut microbiota and metabolites in obesity‐associated chronic gastrointestinal disorders. Nutrients 14, 624. 35276983 10.3390/nu14030624 PMC8838694

[2] Bluher M (2019) Obesity: global epidemiology and pathogenesis. Nat Rev Endocrinol 15, 288–298. 30814686 10.1038/s41574-019-0176-8

[3] de Moura EDM , Dos Reis SA , da Conceição LL , Sediyama CMNO , Pereira SS , de Oliveira LL , Gouveia Peluzio MDC , Martinez JA and Milagro FI (2021) Diet‐induced obesity in animal models: points to consider and influence on metabolic markers. Diabetol Metab Syndr 13, 32. 33736684 10.1186/s13098-021-00647-2 PMC7976703

[4] Golay A and Bobbioni E (1997) The role of dietary fat in obesity. Int J Obes Relat Metab Disord 21 (Suppl 3), S2–S11. 9225171

[5] Wu J , Wang K , Wang X , Pang Y and Jiang C (2021) The role of the gut microbiome and its metabolites in metabolic diseases. Protein Cell 12, 360–373. 33346905 10.1007/s13238-020-00814-7 PMC8106557

[6] Wang B , Kong Q , Li X , Zhao J , Zhang H , Chen W and Wang G (2020) A high‐fat diet increases gut microbiota biodiversity and energy expenditure due to nutrient difference. Nutrients 12, 3197. 33092019 10.3390/nu12103197 PMC7589760

[7] Jo JK , Seo SH , Park SE , Kim HW , Kim EJ , Kim JS , Pyo JY , Cho KM , Kwon SJ , Park DH *et al*. (2021) Gut microbiome and metabolome profiles associated with high‐fat diet in mice. Metabolites 11, 482. 34436423 10.3390/metabo11080482 PMC8398001

[8] Chen HH , Tseng YJ , Wang SY , Tsai YS , Chang CS , Kuo TC , Yao WJ , Shieh CC , Wu CH and Kuo PH (2015) The metabolome profiling and pathway analysis in metabolic healthy and abnormal obesity. Int J Obes (Lond) 39, 1241–1248. 25907313 10.1038/ijo.2015.65

[9] Hases L , Archer A , Indukuri R , Birgersson M , Savva C , Korach‐Andre M and Williams C (2020) High‐fat diet and estrogen impacts the colon and its transcriptome in a sex‐dependent manner. Sci Rep 10, 16160. 32999402 10.1038/s41598-020-73166-1 PMC7527340

[10] Shimi G , Pourvali K , Ghorbani A , Nooshin S , Zare KS , Iranirad R and Zand H (2022) Alterations of DNA methylation and expression of genes related to thyroid hormone metabolism in colon epithelium of obese patients. BMC Med Genomics 15, 229. 36320063 10.1186/s12920-022-01387-6 PMC9628115

[11] Qin Y , Roberts JD , Grimm SA , Lih FB , Deterding LJ , Li R , Chrysovergis K and Wade PA (2018) An obesity‐associated gut microbiome reprograms the intestinal epigenome and leads to altered colonic gene expression. Genome Biol 19, 7. 29361968 10.1186/s13059-018-1389-1 PMC5782396

[12] Kim SE , Choo J , Yoon J , Chu JR , Bae YJ , Lee S , Park T and Sung MK (2017) Genome‐wide analysis identifies colonic genes differentially associated with serum leptin and insulin concentrations in C57BL/6J mice fed a high‐fat diet. PLoS One 12, e0171664. 28170448 10.1371/journal.pone.0171664 PMC5295695

[13] Yang W and Cong Y (2021) Gut microbiota‐derived metabolites in the regulation of host immune responses and immune‐related inflammatory diseases. Cell Mol Immunol 18, 866–877. 33707689 10.1038/s41423-021-00661-4 PMC8115644

[14] Wu ZE , Fraser K , Kruger MC , Sequeira IR , Yip W , Lu LW , Plank LD , Murphy R , Cooper GJS , Martin JC *et al*. (2021) Untargeted metabolomics reveals plasma metabolites predictive of ectopic fat in pancreas and liver as assessed by magnetic resonance imaging: the TOFI_Asia study. Int J Obes (Lond) 45, 1844–1854. 33994541 10.1038/s41366-021-00854-x PMC8310794

[15] Bellot P , Braga ES , Omage FB , da Silva Nunes FL , Lima SCVC , Lyra CO , Marchioni DML , Pedrosa LFC , Barbosa F Jr , Tasic L *et al*. (2023) Plasma lipid metabolites as potential biomarkers for identifying individuals at risk of obesity‐induced metabolic complications. Sci Rep 13, 11729. 37474543 10.1038/s41598-023-38703-8 PMC10359283

[16] Pan XF , Chen ZZ , Wang TJ , Shu X , Cai H , Cai Q , Clish CB , Shi X , Zheng W , Gerszten RE *et al*. (2022) Plasma metabolomic signatures of obesity and risk of type 2 diabetes. Obesity (Silver Spring) 30, 2294–2306. 36161775 10.1002/oby.23549 PMC9633360

[17] Ottosson F , Brunkwall L , Ericson U , Nilsson PM , Almgren P , Fernandez C , Melander O and Orho‐Melander M (2018) Connection between BMI‐related plasma metabolite profile and gut microbiota. J Clin Endocrinol Metab 103, 1491–1501. 29409054 10.1210/jc.2017-02114

[18] Nagarajan SR , Cross E , Sanna F and Hodson L (2022) Dysregulation of hepatic metabolism with obesity: factors influencing glucose and lipid metabolism. Proc Nutr Soc 81, 1–11. 34726148 10.1017/S0029665121003761

[19] Siersbaek M , Varticovski L , Yang S , Baek S , Nielsen R , Mandrup S , Hager GL , Chung JH and Grøntved L (2017) High fat diet‐induced changes of mouse hepatic transcription and enhancer activity can be reversed by subsequent weight loss. Sci Rep 7, 40220. 28071704 10.1038/srep40220 PMC5223143

[20] Quintana‐Castro R , Aguirre‐Maldonado I , Soto‐Rodríguez I , Deschamps‐Lago RA , Gruber‐Pagola P , Urbina de Larrea YK , Juárez‐Rivera VE , Ramos‐Manuel LE and Alexander‐Aguilera A (2020) Cd36 gene expression in adipose and hepatic tissue mediates the lipids accumulation in liver of obese rats with sucrose‐induced hepatic steatosis. Prostaglandins Other Lipid Mediat 147, 106404. 31838198 10.1016/j.prostaglandins.2019.106404

[21] Ma Y , Kan C , Qiu H , Liu Y , Hou N , Han F , Shi J and Sun X (2021) Transcriptomic analysis reveals the protective effects of empagliflozin on lipid metabolism in nonalcoholic fatty liver disease. Front Pharmacol 12, 793586. 34992540 10.3389/fphar.2021.793586 PMC8724565

[22] Wang B , Zhang S , Wang X , Yang S , Jiang Q , Xu Y and Xia W (2017) Transcriptome analysis of the effects of chitosan on the hyperlipidemia and oxidative stress in high‐fat diet fed mice. Int J Biol Macromol 102, 104–110. 28385522 10.1016/j.ijbiomac.2017.03.187

[23] Chen Q , Xiong C , Jia K , Jin J , Li Z , Huang Y , Liu Y , Wang L , Luo H , Li H *et al*. (2019) Hepatic transcriptome analysis from HFD‐fed mice defines a long noncoding RNA regulating cellular cholesterol levels. J Lipid Res 60, 341–352. 30504232 10.1194/jlr.M086215 PMC6358296

[24] Qin J , Li Y , Cai Z , Li S , Zhu J , Zhang F , Liang S , Zhang W , Guan Y , Shen D *et al*. (2012) A metagenome‐wide association study of gut microbiota in type 2 diabetes. Nature 490, 55–60. 23023125 10.1038/nature11450

[25] Beghini F , McIver LJ , Blanco‐Míguez A , Dubois L , Asnicar F , Maharjan S , Mailyan A , Manghi P , Scholz M , Thomas AM *et al*. (2021) Integrating taxonomic, functional, and strain‐level profiling of diverse microbial communities with bioBakery 3. Elife 10, e65088. 33944776 10.7554/eLife.65088 PMC8096432

[26] Sonnenburg JL and Backhed F (2016) Diet‐microbiota interactions as moderators of human metabolism. Nature 535, 56–64. 27383980 10.1038/nature18846 PMC5991619

[27] Gomes AC , Hoffmann C and Mota JF (2018) The human gut microbiota: metabolism and perspective in obesity. Gut Microbes 9, 308–325. 29667480 10.1080/19490976.2018.1465157 PMC6219651

[28] Stanislawski MA , Dabelea D , Lange LA , Wagner BD and Lozupone CA (2019) Gut microbiota phenotypes of obesity. NPJ Biofilms Microbiomes 5, 18. 31285833 10.1038/s41522-019-0091-8 PMC6603011

[29] Liu Y , Yang K , Jia Y , Shi J , Tong Z , Fang D , Yang B , Su C , Li R , Xiao X *et al*. (2021) Gut microbiome alterations in high‐fat‐diet‐fed mice are associated with antibiotic tolerance. Nat Microbiol 6, 874–884. 34017107 10.1038/s41564-021-00912-0

[30] Magne F , Gotteland M , Gauthier L , Zazueta A , Pesoa S , Navarrete P and Balamurugan R (2020) The firmicutes/bacteroidetes ratio: a relevant marker of gut dysbiosis in obese patients? Nutrients 12, 1474. 32438689 10.3390/nu12051474 PMC7285218

[31] Zhang Q , Kim JH , Kim Y and Kim W (2020) *Lactococcus chungangensis* CAU 28 alleviates diet‐induced obesity and adipose tissue metabolism in vitro and in mice fed a high‐fat diet. J Dairy Sci 103, 9803–9814. 32896398 10.3168/jds.2020-18681

[32] Zhu K , Tan F , Mu J , Yi R , Zhou X and Zhao X (2019) Anti‐obesity effects of lactobacillus fermentum CQPC05 isolated from Sichuan pickle in high‐fat diet‐induced obese mice through PPAR‐alpha signaling pathway. Microorganisms 7, 194. 31284674 10.3390/microorganisms7070194 PMC6680547

[33] Chelladhurai K , Ayyash M , Turner MS and Kamal‐Eldin A (2023) *Lactobacillus helveticus*: health effects, current applications, and future trends in dairy fermentation. Trends Food Sci Technol 136, 159–168.

[34] Bailen M , Bressa C , Martinez‐Lopez S , Gonzalez‐Soltero R , Montalvo LM , San JC and Larrosa M (2020) Microbiota features associated with a high‐fat/low‐fiber diet in healthy adults. Front Nutr 7, 583608. 33392236 10.3389/fnut.2020.583608 PMC7775391

[35] Pham MT , Yang JJ , Balasubramaniam A , Rahim AR , Adi P , Do T , Herr DR and Huang CM (2020) *Leuconostoc mesenteroides* mediates an electrogenic pathway to attenuate the accumulation of abdominal fat mass induced by high fat diet. Sci Rep 10, 21916. 33318546 10.1038/s41598-020-78835-9 PMC7736347

[36] Squillario M , Bonaretti C , La Valle A , Di Marco E , Piccolo G , Minuto N , Patti G , Napoli F , Bassi M , Maghnie M *et al*. (2023) Gut‐microbiota in children and adolescents with obesity: inferred functional analysis and machine‐learning algorithms to classify microorganisms. Sci Rep 13, 11294. 37438382 10.1038/s41598-023-36533-2 PMC10338520

[37] Wu TR , Lin CS , Chang CJ , Lin TL , Martel J , Ko YF , Ojcius DM , Lu CC , Young JD and Lai HC (2019) Gut commensal *Parabacteroides goldsteinii* plays a predominant role in the anti‐obesity effects of polysaccharides isolated from *Hirsutella sinensis* . Gut 68, 248–262. 30007918 10.1136/gutjnl-2017-315458

[38] Wei S , Wang J , Wang C , Wang Y and Jin M (2022) Inulin mitigates high fructose‐induced gut dysbiosis and metabolic dysfunction in mice. J Funct Foods 97, 105236.

[39] Henneke L , Schlicht K , Andreani NA , Hollstein T , Demetrowitsch T , Knappe C , Hartmann K , Jensen‐Kroll J , Rohmann N , Pohlschneider D *et al*. (2022) A dietary carbohydrate – gut *Parasutterella* – human fatty acid biosynthesis metabolic axis in obesity and type 2 diabetes. Gut Microbes 14, 2057778. 35435797 10.1080/19490976.2022.2057778 PMC9037427

[40] Seo KH , Gyu LH , Young EJ , Jin JH , Yokoyama W and Kim H (2022) Effects of kefir lactic acid bacteria‐derived postbiotic components on high fat diet‐induced gut microbiota and obesity. Food Res Int 157, 111445. 35761685 10.1016/j.foodres.2022.111445

[41] Oluwagbemigun K , Anesi A , Ulaszewska M , Clarke G , Alexy U , Schmid M , Roden M , Herder C , Mattivi F and Nöthlings U (2020) Longitudinal relationship of amino acids and indole metabolites with long‐term body mass index and cardiometabolic risk markers in young individuals. Sci Rep 10, 6399. 32286421 10.1038/s41598-020-63313-z PMC7156759

[42] Su Y , Feng Z , He Y , Hong L , Liu G , Li T and Yin Y (2019) Monosodium L‐glutamate and fats change free fatty acid concentrations in intestinal contents and affect free fatty acid receptors express profile in growing pigs. Food Nutr Res 63, doi: 10.29219/fnr.v63.1444 PMC6642617 31360149

[43] Fan Y and Pedersen O (2021) Gut microbiota in human metabolic health and disease. Nat Rev Microbiol 19, 55–71. 32887946 10.1038/s41579-020-0433-9

[44] Lischka J , Schanzer A , Baumgartner M , de Gier C , Greber‐Platzer S and Zeyda M (2022) Tryptophan metabolism is associated with BMI and adipose tissue mass and linked to metabolic disease in pediatric obesity. Nutrients 14, 286. 35057467 10.3390/nu14020286 PMC8781866

[45] Song Z , Wang Y , Zhang F , Yao F and Sun C (2019) Calcium signaling pathways: key pathways in the regulation of obesity. Int J Mol Sci 20, 2768. 31195699 10.3390/ijms20112768 PMC6600289

[46] Hildebrandt X , Ibrahim M and Peltzer N (2023) Cell death and inflammation during obesity: “know my methods, WAT(son)”. Cell Death Differ 30, 279–292. 36175539 10.1038/s41418-022-01062-4 PMC9520110

[47] Hagenbeek FA , Pool R , van Dongen J , Draisma HHM , Jan Hottenga J , Willemsen G , Abdellaoui A , Fedko IO , den Braber A , Visser PJ *et al*. (2020) Heritability estimates for 361 blood metabolites across 40 genome‐wide association studies. Nat Commun 11, 39. 31911595 10.1038/s41467-019-13770-6 PMC6946682

[48] Shin SY , Fauman EB , Petersen AK , Krumsiek J , Santos R , Huang J , Arnold M , Erte I , Forgetta V , Yang TP *et al*. (2014) An atlas of genetic influences on human blood metabolites. Nat Genet 46, 543–550. 24816252 10.1038/ng.2982 PMC4064254

[49] Tripathi A , Debelius J , Brenner DA , Karin M , Loomba R , Schnabl B and Knight R (2018) The gut‐liver axis and the intersection with the microbiome. Nat Rev Gastroenterol Hepatol 15, 397–411. 29748586 10.1038/s41575-018-0011-z PMC6319369

[50] Lala V , Zubair M and Minter DA (2023) Liver function tests. In StatPearls. StatPearls Publishing, Treasure Island, FL. 29494096

[51] Zhang Y , Guo S , Xie C and Fang J (2020) Uridine metabolism and its role in glucose, lipid, and amino acid homeostasis. Biomed Res Int 2020, 7091718. 32382566 10.1155/2020/7091718 PMC7180397

[52] Kubota Y , Han Q , Reynoso J , Aoki Y , Masaki N , Obara K , Hamada K , Bouvet M , Tsunoda T and Hoffman RM (2023) Old‐age‐induced obesity reversed by a methionine‐deficient diet or oral administration of recombinant methioninase‐producing *Escherichia coli* in C57BL/6 mice. Aging (Albany NY) 15, 4642–4648. 37301544 10.18632/aging.204783 PMC10292902

[53] Zhen D , Liu J , Zhang XD and Song Z (2022) Kynurenic acid acts as a signaling molecule regulating energy expenditure and is closely associated with metabolic diseases. Front Endocrinol (Lausanne) 13, 847611. 35282457 10.3389/fendo.2022.847611 PMC8908966

[54] Pooyandjoo M , Nouhi M , Shab‐Bidar S , Djafarian K and Olyaeemanesh A (2016) The effect of (L‐)carnitine on weight loss in adults: a systematic review and meta‐analysis of randomized controlled trials. Obes Rev 17, 970–976. 27335245 10.1111/obr.12436

[55] Chung HH , Lee KS and Cheng JT (2013) Decrease of obesity by allantoin via imidazoline I 1‐receptor activation in high fat diet‐fed mice. Evid Based Complement Alternat Med 2013, 589309. 23606885 10.1155/2013/589309 PMC3626183

[56] George C , Barras M , Coombes J and Winckel K (2020) Unfractionated heparin dosing in obese patients. Int J Clin Pharmacol 42, 462–473. 10.1007/s11096-020-01004-5 32140914

[57] Dehghan P , Farhangi MA , Nikniaz L , Nikniaz Z and Asghari‐Jafarabadi M (2020) Gut microbiota‐derived metabolite trimethylamine N‐oxide (TMAO) potentially increases the risk of obesity in adults: an exploratory systematic review and dose‐response meta‐ analysis. Obes Rev 21, e12993. 32017391 10.1111/obr.12993

[58] Alves A , Bassot A , Bulteau AL , Pirola L and Morio B (2019) Glycine metabolism and its alterations in obesity and metabolic diseases. Nutrients 11, 1356. 31208147 10.3390/nu11061356 PMC6627940

[59] Alves A and Morio B (2023) Alterations in glycine metabolism in obesity and chronic metabolic diseases – an update on new advances. Curr Opin Clin Nutr Metab Care 26, 50–54. 36542534 10.1097/MCO.0000000000000883

[60] No authors (2020) Corrigendum. Am J Physiol Endocrinol Metab 319, E458. 37525447 10.1152/ajpendo.zh1-8421-corr.2020 PMC10409521

[61] Zhang B , Jiang M , Zhao J , Song Y , Du W and Shi J (2022) The mechanism underlying the influence of Indole‐3‐propionic acid: a relevance to metabolic disorders. Front Endocrinol (Lausanne) 13, 841703. 35370963 10.3389/fendo.2022.841703 PMC8972051

