# Correction to ‘In‐depth multiomic characterization of the effects of obesity in high‐fat diet‐fed mice’

**DOI:** 10.1002/2211-5463.70095

**Published:** 2025-07-30

Boping Li, Juanjuan Chen, Xiaobin Ou, Xiuli Liu, Zaoxu Xu, Xuesong Xiang, Yan Yang, Qi Wang. In‐depth multiomic characterization of the effects of obesity in high‐fat diet‐fed mice. *FEBS Open Bio*. 2024;14: 771–792. https://doi.org/10.1002/2211‐5463.13788

The Acknowledgements section published in the article is incomplete. The corrected ‘Acknowledgements’ is as follows:

