# Supplementary material for: In‐depth multiomic characterization of the effects of obesity in high‐fat diet‐fed mice
Source: FEBS Open Bio. 2024 Mar 13;14(5):771–92. doi: 10.1002/2211-5463.13788 (PMC11073502; doi:10.1002/2211-5463.13788)
Supplement: Supplementary file 1 — Fig. S1. Changes of the body weight in the high‐fat diet‐fed mice and the control mice during the whole experiment. Fig. S2. Contents of ALT, AST, TC, TG, and FFA in the liver and blood of HFD‐fed mice and controls. Fig. S3. Levels of IL‐6, TNFα, and LPS in the colon, blood, and liver of HFD‐fed mice and controls. Fig. S4. H&E staining of the main organs and morphology of the mice. Fig. S5. Changes of the gut microbiota before and after HFD treatment. Fig. S6. Functional changes of the gut microbiota before and after treated with HFD. Fig. S7. Correlation analysis of the significantly different species and metabolites in Fig. 2D. Fig. S8. Differential analysis of metabolites in the plasma of HFD‐fed and control mice. Fig. S9. Correlation analysis of the colon and plasma metabolites in HFD‐fed mice. Fig. S10. Correlation analysis of the significantly different gut microbial metabolites and common metabolites showed contrary enrichment in colon and plasma between two groups. Fig. S11. Variation analysis of the liver metabolisms. Fig. S12. Venn analysis of metabolites from colonic content, liver and plasma. [file FEB4-14-771-s001.pdf]

Supplementary materials

Supplementary figures

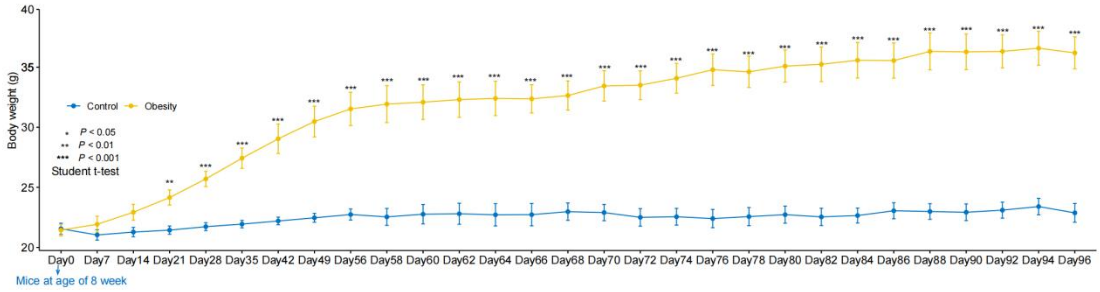

Figure S1. Changes of the body weight in the high-fat diet-fed mice and the control mice during the whole experiment.

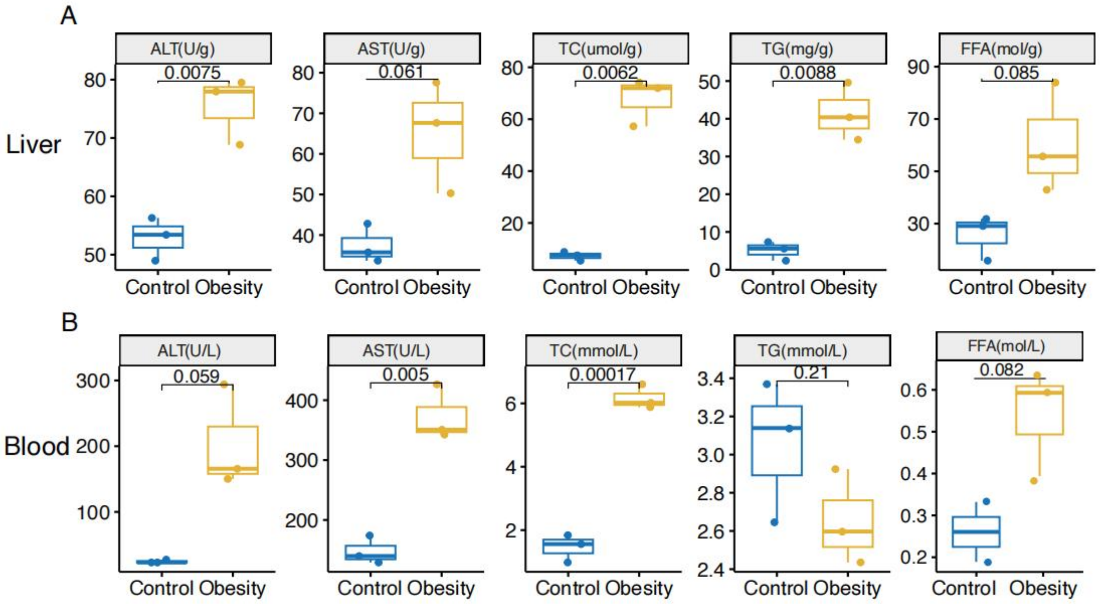

Figure S2. Contents of ALT, AST, TC, TG and FFA in the liver and blood of the obese mice and controls.

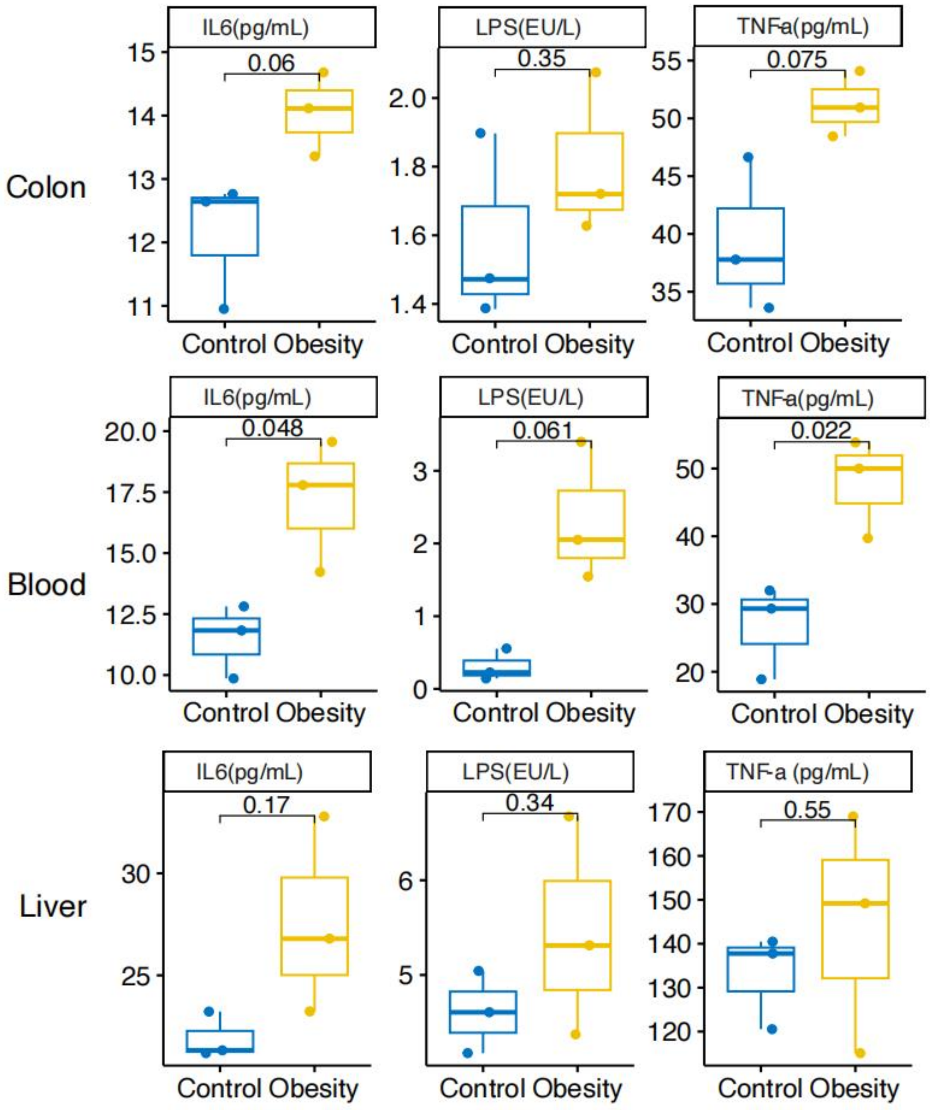

Figure S3. Levels of IL-6, TNFα and LPS in colon, blood and liver of the obese mice and controls.

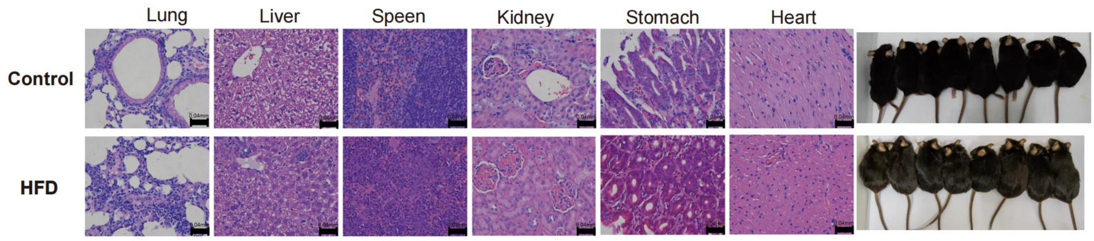

Figure S4. H&E staining of the main organs and the morphological pictures of two groups of mice. H&E staining showed no significant differences of the organs between groups, suggesting no damage of organs was caused by HFD feeding. The morphological pictures showed that the mice fed by HFD were obese compared with control mice. The picture is 400 times enlarged (The pictures was taken under a microscope with a 40× objective and 10× eyepiece) with a scale of 40 μm.

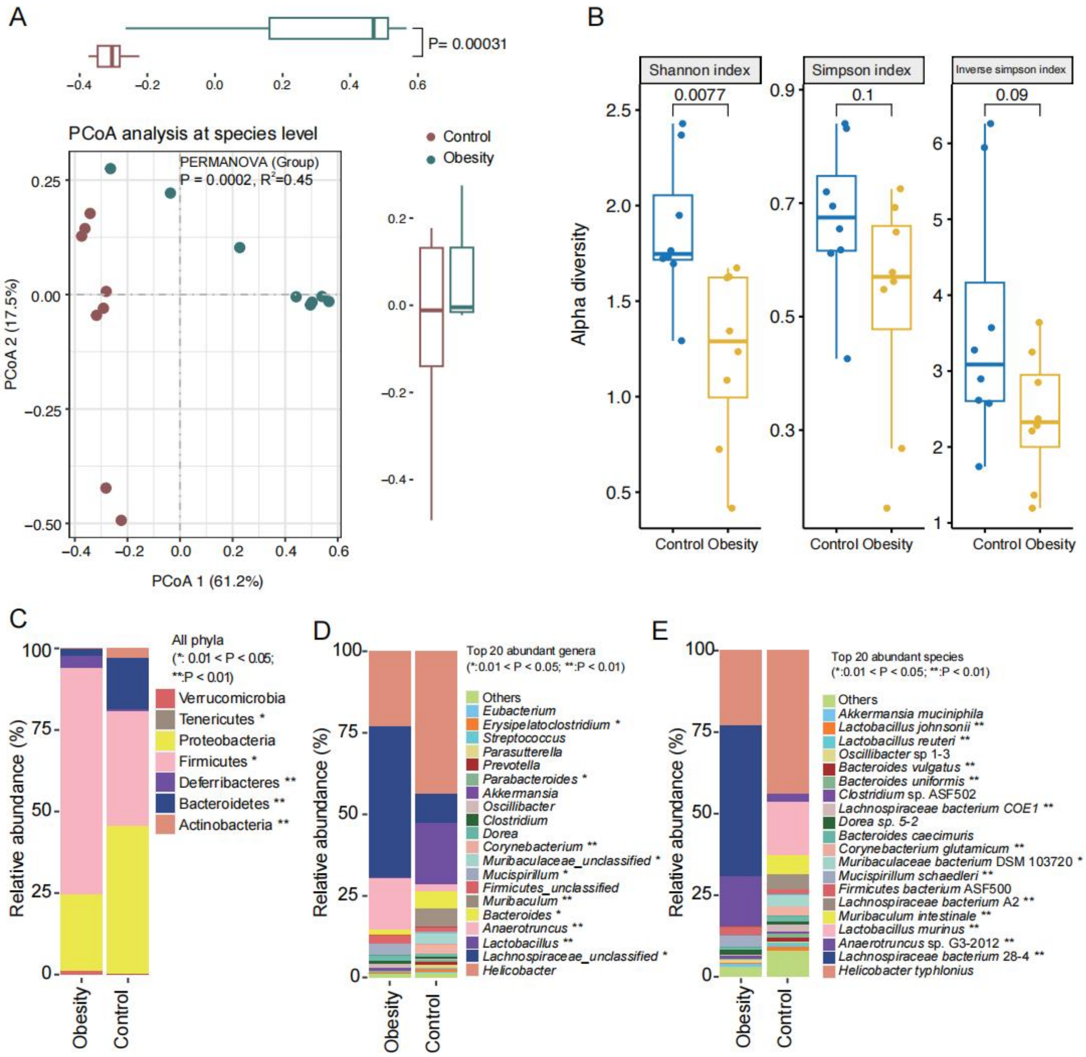

Figure S5. Changes of the gut microbiota before and after HFD treatment. (A) PCoA analysis based on bray-curtis distance at species level. (B) Alpha diversity analysis at species level based on Shannon index, Simpson index, and Inverse Simpson index. (C) Significantly different phyla. (D) Top 20 abundant genera. (E) Top 20 abundant species.

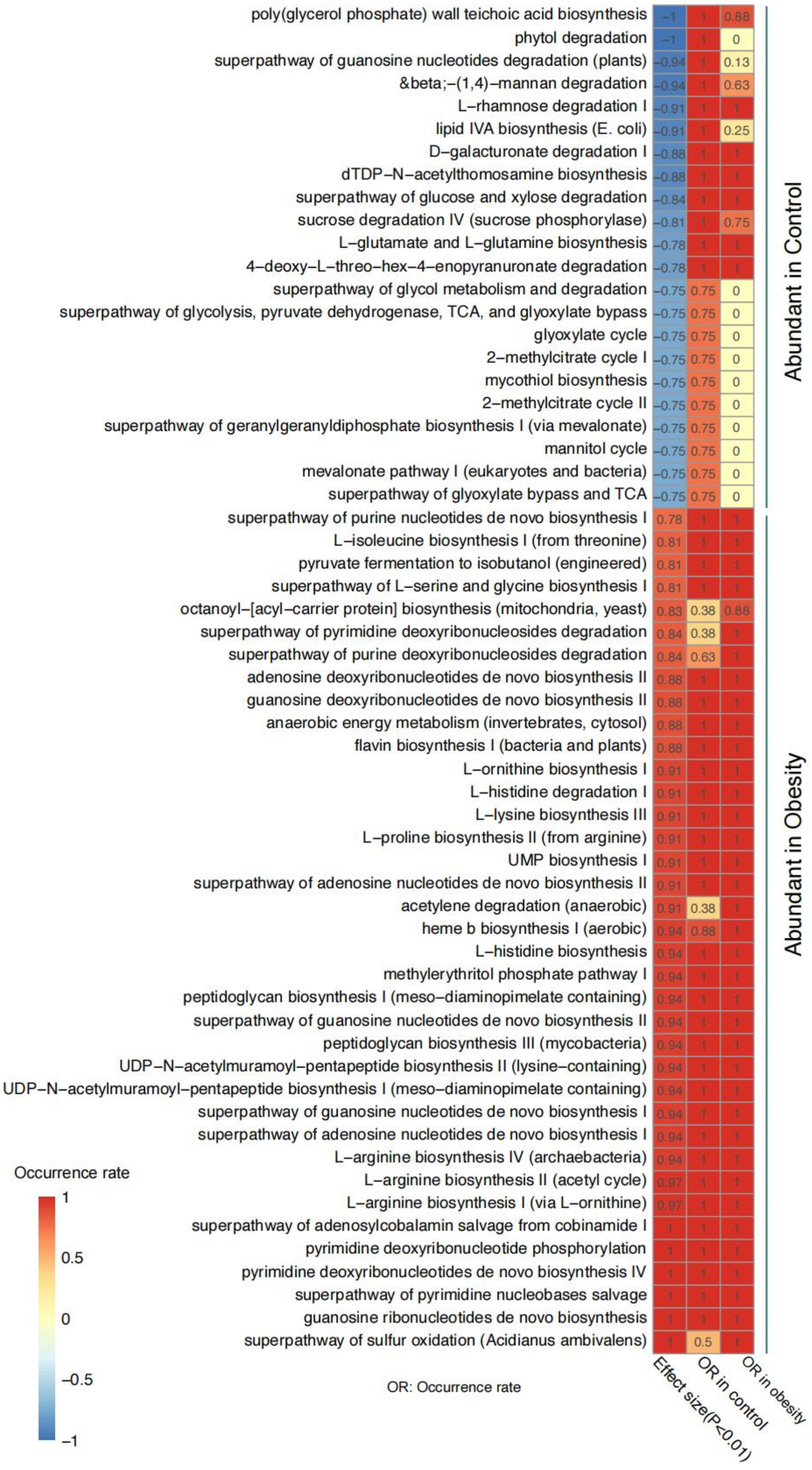

Figure S6. Functional changes of the gut microbiota before and after treated with HFD.

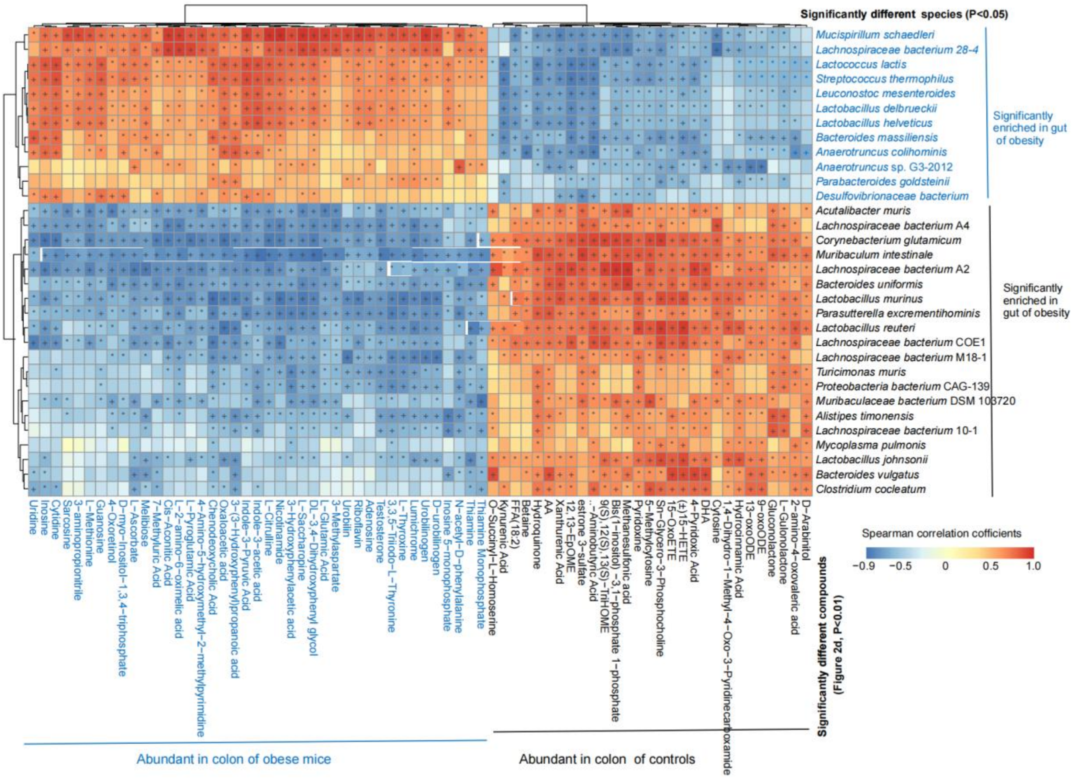

Figure S7. Correlation analysis of the significantly different species and metabolites in Figure 2D.

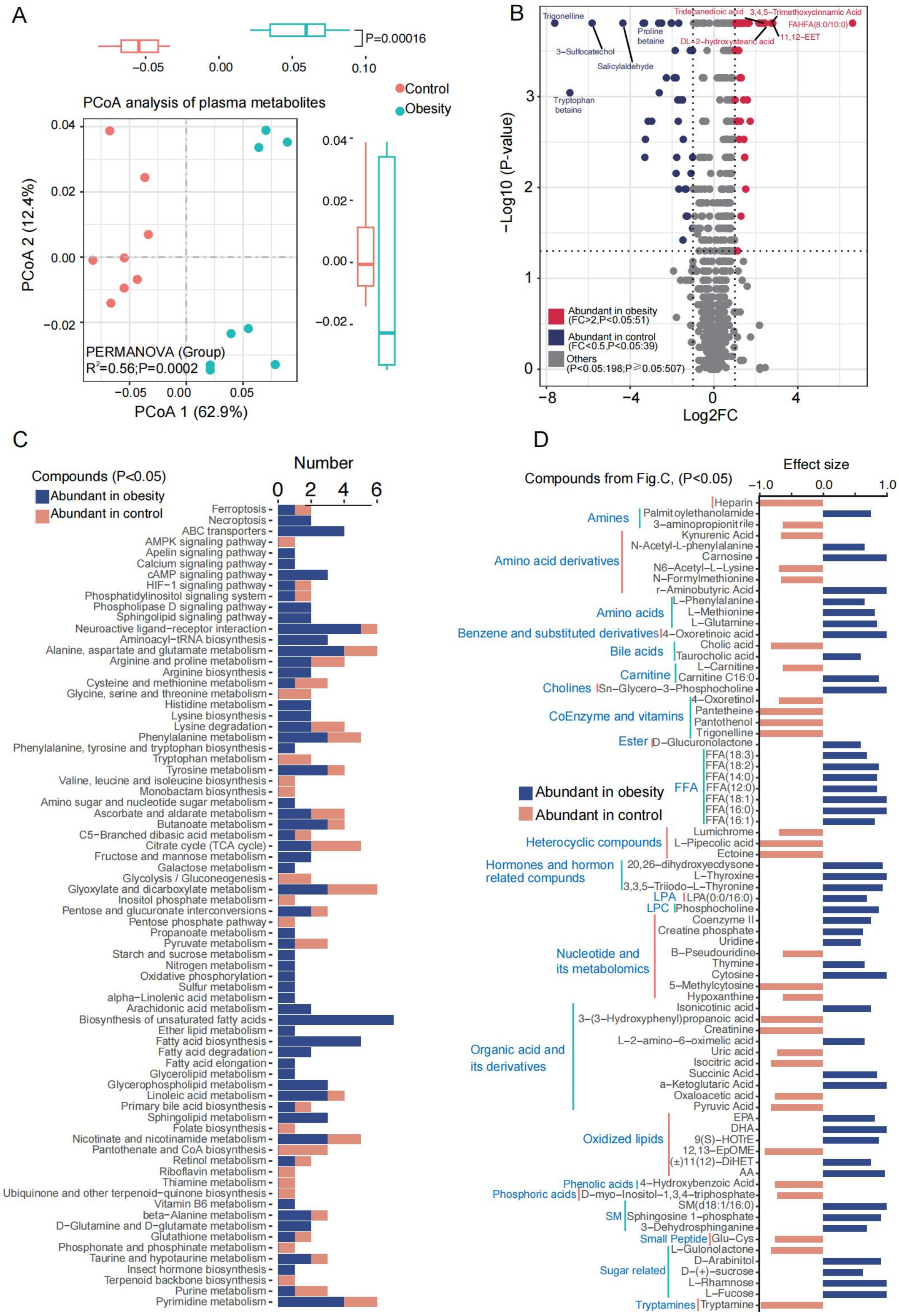

Figure S8. Differential analysis of metabolites in the plasma of obese and control mice. (A) PCoA showing significant differences in plasma metabolites between the two groups. (B) Heatmap showing differences in plasma metabolites between the two groups in the two groups. (C) KEGG pathways associated with the significantly different plasma metabolites. (D) Significantly different plasma metabolites between the two groups.

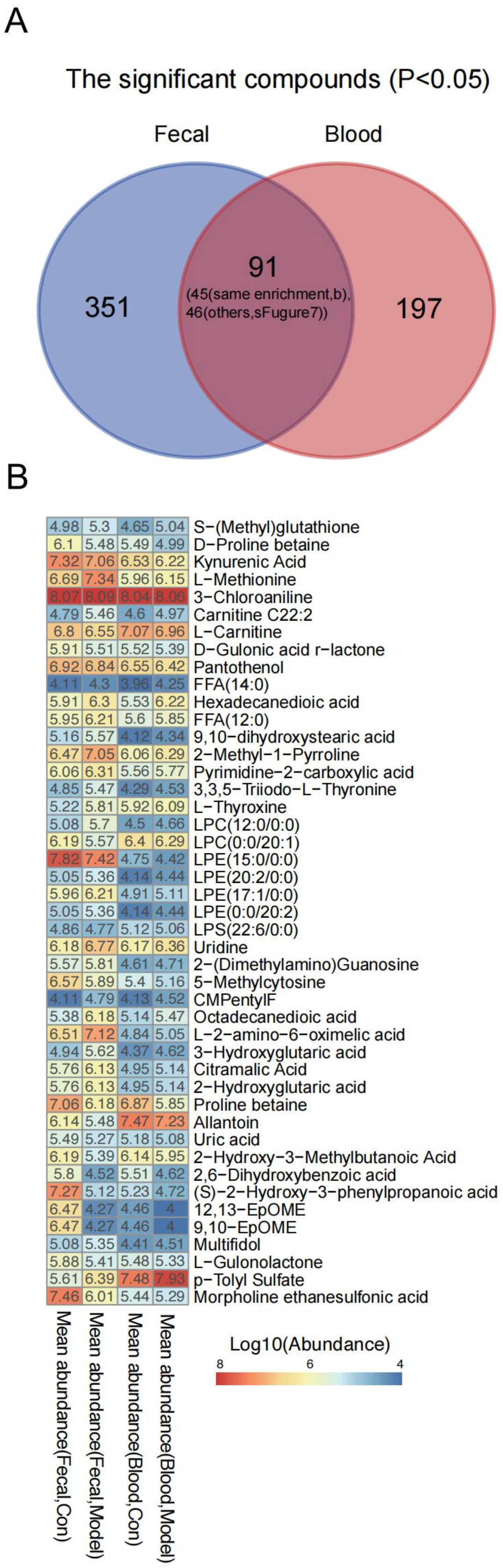

Figure S9. Correlation analysis of the colon and plasma metabolites.

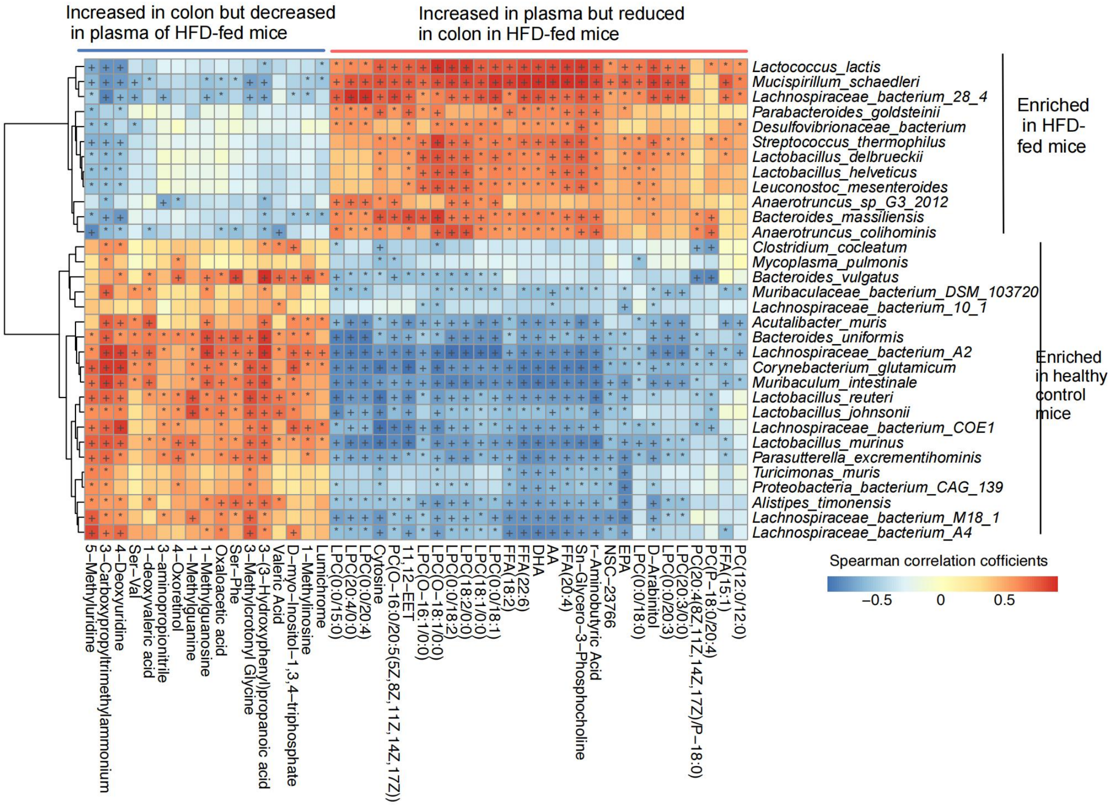

Figure S10. Correlation analysis of the significantly different gut microbial metabolites and common metabolites showed contrary enrichment in colon and plasma between two groups.

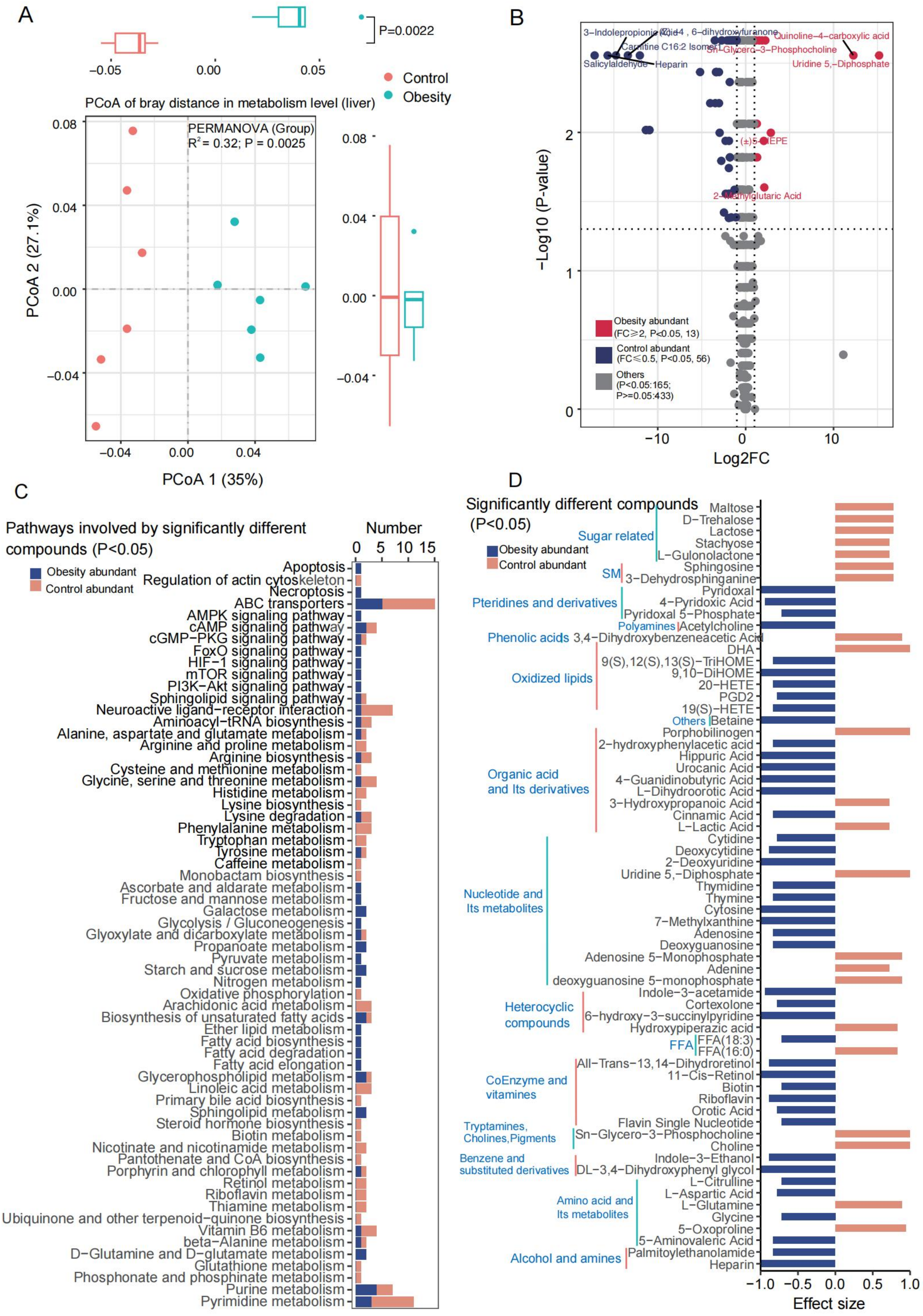

Figure S11. Variation analysis of the liver metabolisms. (A) PCoA showing significant differences in the liver metabolites between obese and control mice. (B) Heatmap showing the differences between the two groups. (C) KEGG pathways associated with the significantly different metabolites in the livers of the two groups. (D) The significantly different liver metabolites between obese and control mice.

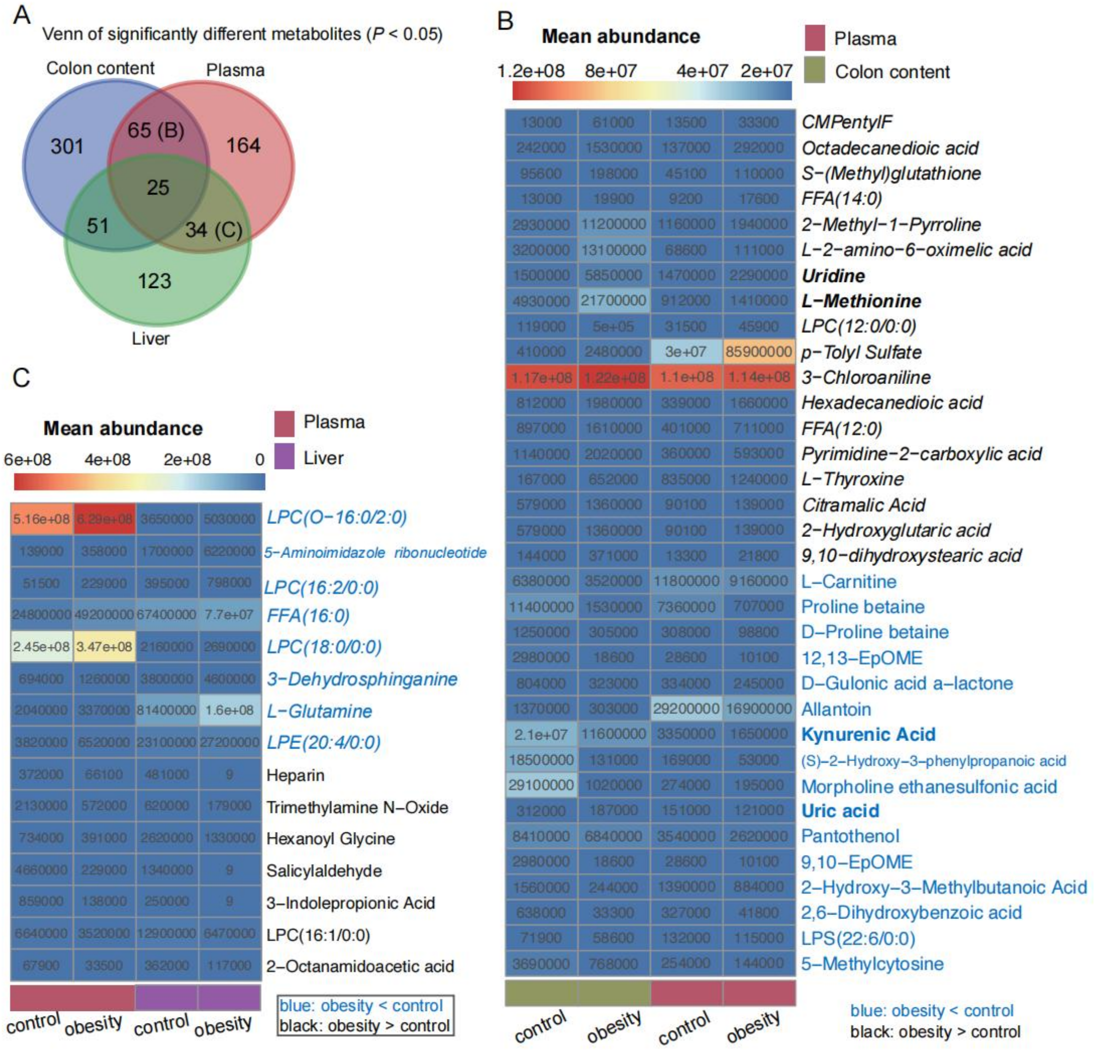

Figure S12. Venn analysis of metabolites from colonic content, liver and plasma.
